# Engineering 3-Hydroxypropionic Acid Production from Glucose in *Yarrowia lipolytica* through Malonyl-CoA Pathway

**DOI:** 10.3390/jof9050573

**Published:** 2023-05-15

**Authors:** Shiyu Liu, Yao Sun, Tianhui Wei, Dianliang Gong, Qi Wang, Zhe Zhan, Jinzhu Song

**Affiliations:** School of Life Science and Technology, Harbin Institute of Technology, Harbin 150006, China; shiyuliu@hit.edu.cn (S.L.); sunyao@stu.hit.edu.cn (Y.S.); weitianhui@stu.hit.edu.cn (T.W.); 18B928017@stu.hit.edu.cn (D.G.); 19B928027@stu.hit.edu.cn (Q.W.); zhanzhe@stu.hit.edu.cn (Z.Z.)

**Keywords:** 3-hydroxypropionic acid, *Yarrowia lipolytica*, malonyl-CoA, degradation

## Abstract

3-Hydroxypropionic acid (3-HP) is an important intermediate compound in the chemical industry. Green and environmentally friendly microbial synthesis methods are becoming increasingly popular in a range of industries. Compared to other chassis cells, *Yarrowia lipolytica* possesses advantages, such as high tolerance to organic acid and a sufficient precursor required to synthesize 3-HP. In this study, gene manipulations, including the overexpression of genes *MCR-N${}_{Ca}$*, *MCR-C${}_{Ca}$*, *GAPN${}_{Sm}$*, *ACC1* and *ACS${}_{Se}^{L641P}$* and knocking out bypass genes *MLS1* and *CIT2*, leading to the glyoxylate cycle, were performed to construct a recombinant strain. Based on this, the degradation pathway of 3-HP in *Y. lipolytica* was discovered, and relevant genes *MMSDH* and *HPDH* were knocked out. To our knowledge, this study is the first to produce 3-HP in *Y. lipolytica*. The yield of 3-HP in recombinant strain Po1f-NC-14 in shake flask fermentation reached 1.128 g·L^−1^, and the yield in fed-batch fermentation reached 16.23 g·L^−1^. These results are highly competitive compared to other yeast chassis cells. This study creates the foundation for the production of 3-HP in *Y. lipolytica* and also provides a reference for further research in the future.

## 1. Introduction

3-Hydroxypropionic acid (3-HP, C_3_H_6_O_3_) was listed as 1 of the 12 kinds of platform chemicals with the greatest potential by the US Department of Energy (DOE) in 2004. It has great application prospects and market value [1]. Many industry chemicals can be synthesized through diverse chemical reactions using 3-HP as material, such as malonic acid, 1,3-propanediol, acrylic acid, β-propiolactone, etc. [2]. Biodegradable plastics, including poly(3-hydroxypropionate) (P3HP) [3] and poly(3-hydroxybutyrate) (PHB) [4], can also be synthesized using 3-HP or its esterified derivatives. They have broad prospects in substituting for traditional petroleum-based plastics. Due to the high demand for 3-HP in the market, a high-yield 3-HP synthesis method is the current research focus. There are some general disadvantages in traditional chemical synthesis methods, such as the high cost of the starting materials and process, as well as the environmental incompatibility of the chemical approaches. Therefore, it is more suitable to obtain 3-HP using a biosynthesis method [5].

Researchers initially tried to synthesize 3-HP using prokaryote as a platform. Because of the clear genetic background and diversified gene manipulation tools, *Escherichia coli* became the first choice for the heterologous expression for production of 3-HP [6]. The Park research group expressed glycerol dehydratase (DHAB) from *Klebsiella pneumonia* and aldehyde dehydrogenase (ALDH) from *Escherichia coli* in *E. coli* BL21, and the initial yield of 3-HP was 0.58 g·L^−1^ by flask fermentation [7]. After optimizing the conditions in terms of pH, medium composition, dissolved oxygen, etc., the concentration of 3-HP reached 31 g·L^−1^ in a fed-batch culture using a 5 L bioreactor [8]. On this basis, the researchers switched the chassis strain to *E. coli* BL21(DE3), which had stronger tolerance to 3-HP and overexpressed glycerol dehydratase (DHAB) and its activators GdrA and GdrB from *K. pneumoniae* and α-ketoglutarate semialdehyde dehydrogenase (KGSADH) from *Azospirillum brasilense*. The yield of 3-HP was increased by 24.8% to 38.7 g·L^−1^ in a fed-batch culture [9]. With the premise of overexpressing the key rate-limiting enzyme, knocking out the bypass pathway genes can also increase the yield of target product. Another research work knocked out *ACKA*, *PTA*, *YQHD*, which are the key genes used to synthesize acetic acid and ethanol after expressing the mutated succinic semialdehyde dehydrogenase (GabD4 _E209Q/E269Q) from *Cupriavidus necator* and glycerol dehydratase (DHAB) from *K. pneumoniae*. The yield of 3-HP reached by 71.9 g·L^−1^ in a fed-batch culture [10], which was the highest yield of 3-HP synthesized in *E. coli* using glycerol as the substrate. Additionally, researchers tried to synthesize 3-HP through the malonyl-CoA pathway in *E. coli*. Malonyl-CoA reductase (MCR) from *Chloroflexus aurantiacus* and acetyl-CoA carboxylase (ACC) from *Corynebacterium glutamicum* were expressed in *E. coli*, and the yield of 3-HP reached 10.08 g·L^−1^ after 36 h fermentation in a fed-batch culture [11]. In order to fundamentally improve the activity of the limiting enzyme, researchers found that the enzyme activity of malonyl-CoA reductase (MCR) from *C. aurantiacus* was severely inhibited under the condition of the bacterial culture temperature. The MCR was split into two different functional structures MCR-N and MCR-C to catalyze reactions individually. Finally, the yield of 3-HP reached 40.6 g·L^−1^ in a fed-batch culture [12]. Comparing to *E. coli*, *K. pneumoniae* has stable glycerol dehydratase (DHAB) and activators; it can also synthesize VB12, which is the key co-enzyme in the glycerol pathway. To explore gene modification from another research direction, researchers overexpressed aldehyde dehydrogenase (ALDH) from *Pseudomonas* sp. and used an appropriate promoter tac. The chassis cell was also knocked out the bypass gene *LDH1*, *LDH2* and *PTA*. The yield of 3-HP reached 83.8 g·L^−1^ 3-HP [13]. They recruited three tandem repetitive *tac* promoters to overexpress an endogenous ALDH (PUUC) and obtained 102.6 g·L^−1^ 3-HP, which was the highest yield reported so far [14].

The most significant problem with the prokaryotic production of 3-HP is that the prokaryotic chassis strain cannot tolerate low pH and product toxicity. Therefore, the researchers started performing the heterologous synthesis of 3-HP with yeast [15]. The researchers expressed the mutated acetyl coenzyme A (acetyl-CoA) carboxylase (ACC_S659A/S1157A) in *Saccharomyces cerevisiae* and preliminarily synthesized 3-HP; the yield was 0.28 g·L^−1^ [16]. On this basis, they expressed a series malonyl-CoA pathway key genes in *S. cerevisiae*, including *MCR${}_{Ca}$*, *ACC1*, *ALD6*, *ACE${}_{SE}$*, *ADH2*, and knocked out *MLS1* which encodes cytosolic malate synthase; the yield of 3-HP reached 0.46 g·L^−1^ [17]. In another study, gene expression cassettes P${}_{TEF1}$BS123-*MCR* and P${}_{HXT1}$-FAS1 were constructed in *S. cerevisiae*. The malonyl-CoA sensor was developed based on the FapR transcription factor of *Bacillus subtilis* and applied in *S. cerevisiae*. The yield of 3-HP reached 0.8 g·L^−1^ [18]. In contrast with conventional metabolic regulation, for a different type of study, researchers designed a malonyl-CoA sensor in *S. cerevisiae* using an adapted bacterial transcription factor *FapR* and its corresponding operator *fapO* to gauge intracellular malonyl-CoA levels. Meanwhile, they co-overexpressed the two novel gene targets *PMP1* and *TPL1*, which were discovered by integrated sensor–genetic screening and achieved a concentration of 3-HP of 1.2 g·L^−1^ [19]. In addition, another research study expressed four heterologous genes in *P. pastoris*, encoding for two functional domains of malonyl-CoA reductase from *C. aurantiacus* (MCR-N, MCR-C), acetyl-CoA carboxylase from *Y. lipolytica* (ACC${}_{Yl}$), and cytosolic NADH kinase from *S. cerevisiae* (cPOS${}_{Sc}$). The recombinant strain was fermented in a 5 L bioreactor by a fed-batch culture, achieving a final concentration of 3-HP of 24.75 g·L^−1^ [20].

*Yarrowia lipolytica*, a representative unconventional yeast, is generally recognized as a safe strain (GRAS), and it can be safely used in supplements for people over 3 years old [21]. As an excellent heterologous expression chassis strain, *Y. lipolytica* synthesizes multiple metabolites, including organic acids, lipases, fatty acids, terpenoids, etc. [22]. Prof. Catherine Madzak modified *Y. lipolytica* (W29, ATCC20460), obtaining Po1d series strains (Po1d, Po1e, Po1f, Po1g, and Po1h), which knocked out the alkaline protease coding gene *AEP* and integrated gene *SUC* in the Po1d strain, which can cause the strain to use sucrose as substrate. Based on the Po1d strain, Po1f strain knocked out the acid protease coding gene *AXP* and eliminated the adverse effect of extracellular protease on the expression of the foreign protein [23,24,25]. In addition, Prof. Catherine Madzak also developed a series of pINA plasmids, including pINA1312 and pINA1269 [26,27]. The strong promoter hp4d was used in the pINA plasmids; it is a growth-dependent promoter that needs no induction and is scarcely affected by the environment, allowing the foreign protein to be expressed in the early stationary phase of cell growth [28]. To increase the efficiency of plasmid integration, the researchers added a zeta sequence to the plasmid. The function of the zeta sequence is that it improves the efficiency of foreign DNA integration into the genome of *Y. lipolytica* (*Ylt1*Δ) by non-homologous end joining (NHEJ) and provides a new way to integrate into the genome of *Y. lipolytica* by NHEJ [29].

**Figure 1:**
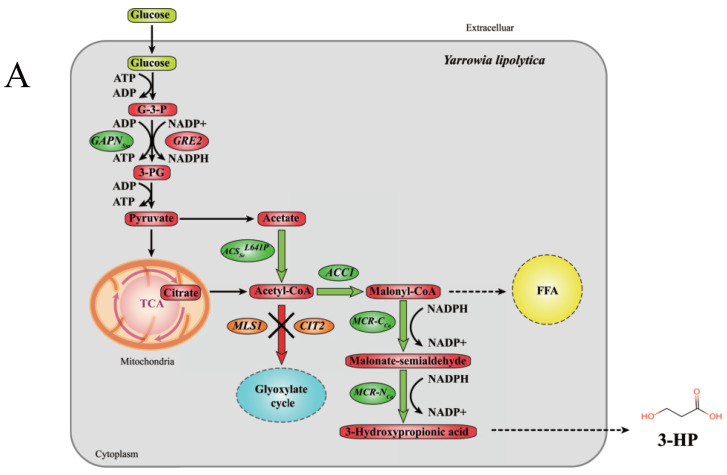

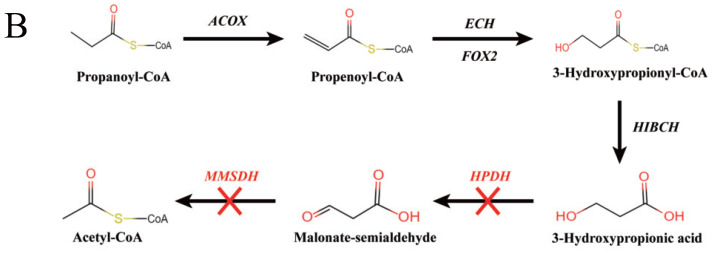
(**A**) Schematic representation of the conversion of glucose to 3-HP through the malonyl-CoA pathway in *Yarrowia lipolytica*. *GRE2*: endogenous NADPH-dependent methylglyoxal reductase coding gene; *GAPN${}_{Sm}$*: non-phosphorylating glyceraldehyde-3-phosphate dehydrogenase coding genefrom *Streptococcus mutans*; *ACS${}_{Se}^{L641P}$*: modified acetyl-CoA synthetase (mutated Leu-641 to Pro) coding gene from *Salmonella enterica*; *ACC1*: acetyl-CoA carboxylase 1 coding gene; *MCR-C${}_{Ca}$*: C-terminal domain of malonyl-CoA reductase coding gene from *Chloroflexus aurantiacus*; *MCR-N${}_{Ca}$*: N-terminal domain of malonyl-CoA reductase coding gene from *Chloroflexus aurantiacus*. All expressed genes are marked by green ellipse. The knockout genes are marked by orange ellipse. *MLS1*: malate synthase 1 coding gene; *CIT2*: citrate synthase 2 coding gene; G-3-P: glyceraldehyde 3-phosphate; 3-PG: 3-phosphoglyceric acid; FFA: free fatty acids. (**B**) Malonate semialdehyde pathway in *Yarrowia lipolytica*. *ACOX*: acyl-CoA oxidase coding gene; *ECH*: enoyl-CoA hydratase coding gene; *FOX2*: multifunctional beta-oxidation protein coding gene; *HIBCH*: 3-hydroxyisobutyryl-CoA hydrolase coding gene; *MMSDH*: methylmalonate-semialdehyde dehydrogenase coding gene; *HPDH*: 3-hydroxypropionate dehydrogenase coding gene. The knockout genes are marked by red cross.

In this study, the production of 3-HP using glucose as substrate was obtained in *Y. lipolytica* through the malonyl-CoA pathway (Figure 1A). We introduced a series of exogenous genes into this pathway, including *MCR* from *C. aurantiacus*, *ACS${}^{L641P}$* from *Salmonella enterica*, and overexpressed endogenous *ACC1*. Meanwhile, the malonyl-CoA reductase was separated into two subunits, which showed higher 3-HP production. Further, *GAPN* from *Streptococcus mutans* was overexpressed to supply NADPH to the system, and the extra added pyruvic acid provided more precursors to the malonyl-CoA pathway. In addition, in order to ensure more fluxes to 3-HP, we knocked out genes *MLS1* and *CIT2*, which introduced more fluxes into the glyoxylate cycle. To solve resolve the 3-HP degradation, we also tried to break the malonate semialdehyde pathway in *Y. lipolytica* by knocking out genes *HPDH* and *MMSDH*, leading to greater accumulation of 3-HP (Figure 1B). Overall, the potential of *Y. lipolytica* as a promising chassis strain for 3-HP production was demonstrated for the first time. In comparison to prokaryotic chassis cells, *Y. lipolytica* has a more suitable fermentation environment for 3-HP (pKa: 4.5) and is more tolerant to the toxicity effects of 3-HP. Our study also provides new insights for increasing the production of 3-HP in the future.

## 2. Materials and Methods

### 2.1. Strain and Plasmid Construction

The *E.coli* strain JM109 was used for plasmid construction and propagation in this study. The auxotrophic *Y. lipolytica* strain Po1f (MATa, Leu2^−^, Ura3^−^; Catherine Madzak, Paris-Saclay University, INRA, AgroParisTech, UMR SayFood, Palaiseau, France) was used as the initial strain. All plasmids used in this study are listed in Table 1, and all recombinant yeast strains were derived from the Po1f strain (Table 2). The plasmids were digested by a restriction enzyme and connected using a ClonExpress Ultra One Step Cloning Kit. All expression cassette were assembled using a strong constitutive hp4d promoter and XPR2 terminator. The overexpression of genes was carried out using the pINA1312 plasmid with URA3ex as a marker and hp4d as a promoter. The expression of genes with diverse copies was carried out using the pINA1269 plasmid with LEU2ex as a marker and hp4d as a promoter. In particular, to increase the translation efficiency in *Y. lipolytica*, we constructed a CACA*ATG* sequence in the gene expression cassettes by adding an “A” before the first codon of the protein of interest (ATG) after digestion by *Pml*I [30]. All sequences of genes investigated in this study are shown in Table S1.

CRISPR/Cas9 vector (JME4580) with HPHex was used to knock out relevant bypass genes [31]. The origin vector was digested by *Bsm*BI to cut off the *E. coli* red fluorescent protein chromophore (RFP) gene and replace it using a sgRNA sequence, which was designed in CRISPOR (version 5.01). All sgRNAs and relevant information are shown in Table S2. The positive transformants can be selected by the color of the colony.

**Table 2:** Strains used in this study.

Strain	Description	Reference
*E. coli* JM109	For construction of recombinant plasmids	Vazyme
*Y. lipolytica* Po1f	MATa, Leu2^−^, Ura3^−^	[32]
Po1f-wMCR	Po1f cells harboring p1312-wMCR	this study
Po1f-tMCR	Po1f cells harboring p1312-tMCR	this study
Po1f-NC-00	Po1f cells harboring p1312-tMCRN-tMCRC	this study
Po1f-NC-01	Po1f cells harboring p1312-NC-*GAPN${}_{Bc}$*	this study
Po1f-NC-02	Po1f cells harboring p1312-NC-*GAPN${}_{Sm}$*	this study
Po1f-NC-03	Po1f cells harboring p1312-NC-*GAPN${}_{Sm}$* and p1269-ACC1	this study
Po1f-NC-04	Po1f cells harboring p1312-NC-*GAPN${}_{Sm}$* and p1269-ACC1-ACC1	this study
Po1f-NC-05	Po1f cells harboring p1312-NC-*GAPN${}_{Sm}$* and p1269-ALD6	this study
Po1f-NC-06	Po1f cells harboring p1312-NC-*GAPN${}_{Sm}$* and p1269-*ACS${}_{Se}^{L641P}$*	this study
Po1f-NC-07	Po1f cells harboring p1312-NC-*GAPN${}_{Sm}$* and p1269-*ACS${}_{Se}^{L641P}$*-*ACS${}_{Se}^{L641P}$*	this study
Po1f-NC-08	Po1f cells harboring p1312-NC-*GAPN${}_{Sm}$* and p1269-ACC1-*ACS${}_{Se}^{L641P}$*	this study
Po1f-NC-09	Po1f-NC-08 transformed with JME4580-MLS1-sgRNA and fragment of donor-MLS1	this study
Po1f-NC-10	Po1f-NC-08 transformed with JME4580-CIT2-sgRNA and fragment of donor-CIT2	this study
Po1f-NC-11	Po1f-NC-09 transformed with JME4580-CIT2-sgRNA and fragment of donor-CIT2	this study
Po1f-NC-12	Po1f-NC-11 transformed with JME4580-HPDH-sgRNA and fragment of donor-HPDH	this study
Po1f-NC-13	Po1f-NC-11 transformed with JME4580-MMSDH-sgRNA and fragment of donor-MMSDH	this study
Po1f-NC-14	Po1f-NC-12 transformed with JME4580-MMSDH-sgRNA and fragment of donor-MMSDH	this study

*Y. lipolytica* transformation was carried out by ZYMO Frozen-EZ yeast transformation II kit [33]. Different types of yeast expression recombinant transformants were selected on SD-Ura and SD-Leu media. The knockout system included a 500 ng CRISPR/Cas9 plasmid and a 500 ng donor fragment. CRISPR/Cas9 deletion transformants were selected on a YPD-hygromycin B medium plate. After verification, the transformants were transferred into YPD media for several generations to allow plasmid curing and were selected on YPD media for the next gene manipulation.

### 2.2. Materials and Yeast Cultivation

*E. coli* with recombinant plasmid was cultivated in Luria Bertani (LB) medium containing 10 g·L^−1^ of tryptone, 5 g·L^−1^ of yeast extract and 10 g·L^−1^. Suitable antibiotics were added in the LB medium plate when necessary at the following final concentrations: kanamycin 50 mg·L^−1^, and ampicillin 100 mg·L^−1^. *Y. lipolytica* and recombinant strains were cultivated in a yeast extract peptone dextrose (YPD) medium (20 g·L^−1^ of tryptone, 20·L^−1^ of yeast extract and 20 g·L^−1^ of glucose) or yeast nitrogen base (YNB) medium (6.7 g·L^−1^ of yeast nitrogen base without amino acids, 10 g·L^−1^ of glucose). The appropriate SD minimal medium was used to select positive yeast transformants such that suitable nutrients were added to the YNB medium plate at the following final concentrations: uracil 0.1 g·L^−1^ and leucine 0.1 g·L^−1^. Hygromycin B (250 mg·L^−1^) was added to the YPD medium plate to select CRISPR/Cas9 deletion transformants. Next, 1.5 g·L^−1^ of agar was added to the solid medium. The genome DNA of yeast was extracted via the CTAB method after grinding with liquid nitrogen. The plasmid extraction kit, first-strand cDNA synthesis kit, SYBR qpcr master mix kit, gel extraction kit, one-step cloning kit and DNA purification kit were all from Vazyme Biotech Co., Ltd. (Nanjing, China). Primer synthesis and Sanger sequencing were performed by Comate Bioscience Co., Ltd. (Changchun, China). All genes were codon optimized and synthesized by General Biosystems (Anhui) Co., Ltd. (Chuzhou, China). All culture media and relevant reagents were purchased from Biosharp Life sciences Co., Ltd. (Hefei, China).

*E. coli* was cultured by LB medium at 37 °C and 180 rpm overnight for plasmid construction and propagation. *Y. lipolytica* strains were cultured by YPD medium, and recombinant strains were cultured by the corresponding SD minimal medium. All strains were incubated in 5 mL medium and grown at 30 °C, 220 rpm to reach an OD${}_{600}$ of 1.0. After that, an initial OD${}_{600}$ of 0.01 yeast was added to 50 mL medium and cultured at 30 °C 220 rpm for 5 days. All the flask fermentation results represent the mean ± S.D. of three independent experiments. CRISPR/Cas9 gene deletion transformants were selected on the YPD-hyg solid medium, and plasmid curing was carried out by culturing in YPD medium for several generations. After that, the transformants were used to perform the next gene knockout manipulation. In addition, 2 g·L^−1^, 4 g·L^−1^, and 6 g·L^−1^ of pyruvic acid were added to the medium of strain Po1f-NC-14 to increase the production of 3-HP by adding a precursor.

### 2.3. Enzyme Assay

The recombinant strains Po1f, Po1f-tMCR and Po1f-NC-00 were cultured in an appropriate SD minimal medium and harvested in the mid-exponential phase. Cells were collected and washed with pre-cooled 0.01 M phosphate-buffered saline (PBS, pH 7.4). Meanwhile, cells were resuspended in PBS in a ratio of 1/20 and lysed using Vibra-Cell ultrasonic liquid processors VC505 (Sonics, Newtown, CT, USA). The lysing conditions included an output power of 500 w; total working time of 15 min; single working time of 7 s; and a gap time of 5 s. An ice box was needed in the lysing environment to reduce the temperature and maintain the biological activity of protein. Lysates were centrifuged at 12,000× *g* for 20 min at 4 °C, and the supernatant was transferred to a new tube to measure the concentration of total protein using a BCA protein quantification kit. Malonyl-CoA reductase activity was measured at 30 °C by mixed buffer (0.3 mL), containing 0.3 mM NADPH, 3 mM DTE, 5 mM MgCl_2_, and 50 mM Tris-HCl buffer. Next, 0.3 mM malonyl-CoA was added into the mixed buffer as a substrate, and the reaction started after the supernatant was added. The assay was monitored at 365 nm ($\epsilon_{NADPH}$ = 3400 M^−1^cm^−1^). For MCR${}_{CA}$ and MCR-N/ MCR-C, one unit of enzyme activity was defined as the amount of enzyme that oxidized 2 μmol of NADPH per minute, corresponding to the enzyme amount required to convert 1 μmol of malonyl-CoA to 3-HP. All the experiments were performed in triplicate.

### 2.4. Analytical Methods

The yeast growth curve was drawn according to the OD${}_{600}$ of the fermentation broth at different times. The OD${}_{600}$ of fermentation broth was measured with an ultraviolet spectrophotometer from Aucy scientific instrument Co., Ltd. (Shanghai, China) and diluted to between 0.3 and 0.8 to ensure linearity of the results. Next, 5 mL fermentation broth, which was cultured after 5 days, was added to 10 ml centrifuge tube and dried to a constant weight in an oven at 105 °C after washing with deionized water. The dry cell weight (DCW) (g·L^−1^) was calculated by dividing the cell dry weight by the volume of the fermentation broth.

The concentration of 3-HP was determined by high-performance liquid chromatography (HPLC). Fermentation broth samples were centrifuged at 12,000× *g* for 10 min, and the supernatants were filtered through a 0.22 μm nitrocellulose filter and analyzed by Waters2695 HPLC (Milford, CT, USA) with an Agilent Eclipse Plus C18 column (4.6 × 100 mm, 3.5 μm) and UV detector. The HPLC conditions were as follows: the injection volume was 10 μL; ratio of mobile phase A (0.1% formic acid aqueous solution with pH 2) and mobile phase B (0.1% formic acid acetonitrile solution with pH 3) was 95:5; the constant flow rate was 0.6 mL·min^−1^; and the column temperature was 30 °C. HPLC-MS analysis was determined by Waters ZQ2000 mass spectrometer (MS) with an ion source of ESI, an ion source temperature of 140 °C, an atomization temperature of 350 °C, and a voltage of 3.5 kV. The SBA-40C biosensor (Biology Institute of Shandong Academy of Science, Jinan, China) was used to determine the glucose concentration of the fermentation broth samples. All the experiments were performed in triplicate.

### 2.5. Rna Isolation and Quantitative Real-Time PCR

The total RNA of yeast was isolated and purified via the TRIZOL method. The cDNA was synthesized using the HiScript III 1st cDNA synthesis kit from Vazyme Biotech Co., Ltd. (Nanjing, China). The relative expression level of different copies of genes, including *ACC1*, *ACS${}_{Se}^{L641P}$* and *ALD6*, was calculated using the the relative quantitative 2${}^{-\Delta \Delta Ct}$ method (compared with Po1f). The mRNA derived from actin was used to normalize the relative expression levels as the internal standard. The AceQ Universal SYBR qPCR Master mix (Vazyme Biotech, Nanjing, China) was used to prepare the qPCR reaction system. The experiment was accomplished by the ViiA7 Real-Time PCR instrument (ABI, Carlsbad, CA, USA). All samples were prepared in triplicate to obtain the Ct value.

### 2.6. Fed-Batch Fermentation

The fed-batch fermentation was carried out in a 1 L bioreactor (Biotech-JG, Shanghai, China). First, an initial OD${}_{600}$ of 0.01 of strain Po1f-NC-14 was inoculated into a 250 mL flask which had 50 mL of the YPD medium and was cultured at 30 °C at 220 rpm for 24 h. After that, 1 mL of fermentation broth was inoculated into 100 mL of the YPD medium and cultured in the same conditions for 24 h. The initial fermentation volume was 500 mL, including the medium (50 g·L^−1^ of glucose, 20 g·L^−1^ of tryptone, and 10 g·L^−1^ of yeast extract), and the inoculum volume was 10%. The fermentation condition was set as follows: pH, 6.0; air flux, 1.5 vvm; and stirring rate, 550 rpm. The pH was maintained by adding with5M NaOH and 5M HCl. Additionally, 10× of the YPD medium was added to the bioreactor to ensure that the concentration of glucose in the fermentation broth reached no less than 10 g·L^−1^. The total fed-batch fermentation time was 240 h, and the concentrations of 3-HP, glucose and OD${}_{600}$ were measured every 12 h. The final concentrations were determined by three independent replicates.

## 3. Results

### 3.1. Construction of Malonyl-CoA Pathway for Synthesis of 3-HP in Y. lipolytica

**Figure 2:**
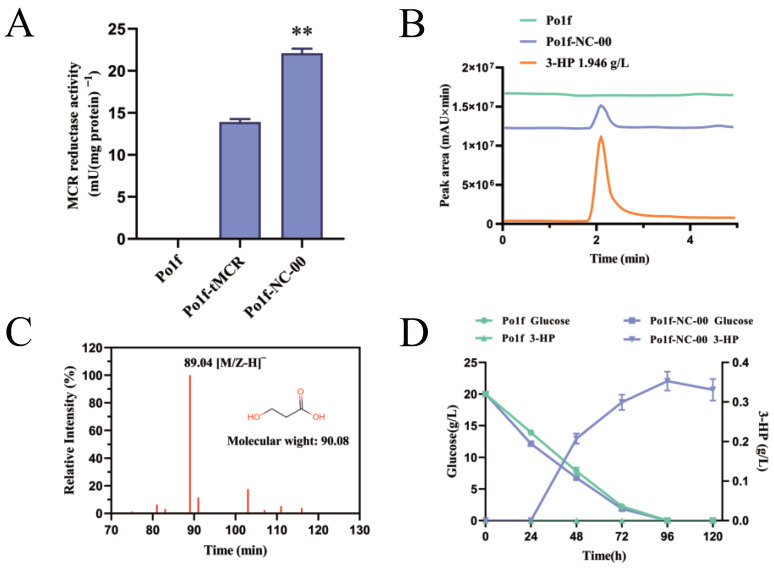
(**A**) Enzyme activity of MCR in strains Po1f, Po1f-tMCR and Po1f-NC-00. One unit of MCR activity was defined as the amount of enzyme required to oxidize 2 mol of NADPH to NADP^+^ per min. Statistical analysis was conducted, with significance indicated by ** *p* < 0.001. (**B**) HPLC analysis of 3-HP product in the supernatant from strains Po1f, Po1f-NC-00 and 3-HP standard sample (1.946 g·L^−1^). (**C**) Identification of 3-HP by HPLC-MS analysis. (**D**) 3-HP accumulation and glucose consumption of strains Po1f and Po1f-NC-00 during 120 h fermentation. Data represent the mean ± SD of biological triplicates in (**A**,**D**).

The genes involved in the conversion of glucose to 3-HP of the malonyl-CoA pathway are shown in Figure 1A. Owing to *Y. lipolytica* having the characteristic of accumulating a large amount of fatty acids by itself, according to the main metabolic pathway, it has a relatively sufficient number of malonyl-CoA precursors [34]. Additionally, *Y. lipolytica* is more tolerant of organic acids [35]. Based on the above two advantages, this study selected *Y. lipolytica* as the chassis cell to synthesize 3-HP through the malonyl-CoA pathway from glucose. According to the literature, we introduced *MCR* from *C. aurantiacus* into *Y. lipolytica* to construct the basic pathway, including the codon-optimized *MCR* gene. More notably, in order to improve the activity of the MCR enzyme, we divided the *MCR* gene (AY530019.1) into two segments [36]. The termination codon ‘TAA’ was added at the end of the N-terminal region of the *MCR* DNA sequence, and the starting codon ‘ATG’ was added at the beginning of the C-terminal region of the *MCR* DNA sequence. MCR-N (550aa) and MCR-C (671aa) showed a higher affinity for malonyl-CoA than MCR. Wild-type *wMCR* from pTrc99a-wMCR, codon-optimized *tMCR*, codon-optimized *MCR-N* and *MCR-C* were all separately connected between the hp4d promoter and XPR2 terminator in pINA1312. After that, p1312-wMCR, p1312-tMCR and p1312-tMCRN-tMCRC were separately transformed into the genome of *Y. lipolytica* to obtain Po1f-wMCR, Po1f-tMCR and Po1f-NC-00. To explore the MCR enzyme activity of the three types of recombinant strains, they were tested using the enzyme assay method. The enzyme activity result in Figure 2A shows that Po1f-NC-00 had much greater MCR enzyme activity (22 mU·mg^−1^) than Po1f-tMCR (14 mU·mg^−1^). Meanwhile, Po1f-wMCR did not exhibit corresponding enzyme activity. It can be inferred that gene *MCR* from *C. aurantiacus* cannot be directly expressed in *Y. lipolytica* without codon optimization. On this basis, we found that splitting the MCR enzyme into two subunits can significantly improve the enzyme activity.

In order to qualitatively and quantitatively analyze the production of 3-HP in the Po1f strain and recombinant strain fermentation broth, Po1f, Po1f-wMCR, Po1f-tMCR and Po1f-NC-00 strains were cultured in YPD, and the fermentation broth samples were analyzed by the modified HPLC method after centrifuging and filtering. The identification of 3-HP in fermentation broth was accomplished using the HPLC-MS method. The results are shown in Figure 2B, where HPLC analysis finds a peak that coincides with the standard sample (1.946 g·L^−1^) at a retention time of 2.1 min. Meanwhile, the result of HPLC-MS analysis in Figure 2C shows that the compound has a molecular ion at *m*/*z*-H 89.04 under the negative ion monitoring mode, which is identical to that of 3-HP.

In order to determine the most suitable fermentation time for 3-HP accumulation and find the relationship between 3-HP production and glucose in the fermentation broth, Po1f and Po1f-NC-00 strains were cultured in YPD (glucose concentration: 20 g·L^−1^) at 30 °C, 220 rpm in a 250 mL flask for 120 h. The concentrations of 3-HP and glucose in fermentation broth were determined every 24 h. The results in Figure 2D show that the glucose consumption rates of Po1f and Po1f-NC-00 strains are relatively close. In the early stage of fermentation, the glucose consumption rate of Po1f-NC-00 strain was slightly faster than Po1f, possibly due to the supplementation of the URA3 auxotroph marker to the genome of *Y. lipolytica*, which made its growth rate faster than that of Po1f strain. During the first 24 h of fermentation, Po1f-NC-00 cannot produce 3-HP. The concentration of 3-HP in the fermentation broth reached its maximum at 96 h, and 3-HP degradation occurred after 96 h. This indicates that 3-HP production stopped as glucose was depleted, while the initial strain of Po1f could not produce 3-HP. Therefore, we chose 96 h as the end time for fermentation to measure the concentration of 3-HP in the fermentation broth of Po1f-wMCR, Po1f-tMCR and Po1f-NC-00. In the Po1f-tMCR strain, 0.203 g·L^−1^ of 3-HP (DCW: 27.93 mg·g^−1^) was accumulated . The concentration of 3-HP reached 0.353 g·L^−1^ (DCW: 46.68 mg·g^−1^) in the Po1f-NC-00 strain. Splitting the MCR into two parts improved the 3-HP titer by 1.74-fold; therefore, we chose to modify the pathway based on the Po1f-NC-00 strain.

### 3.2. Optimization of NADPH Supply to Increase 3-HP Production

**Figure 3:**
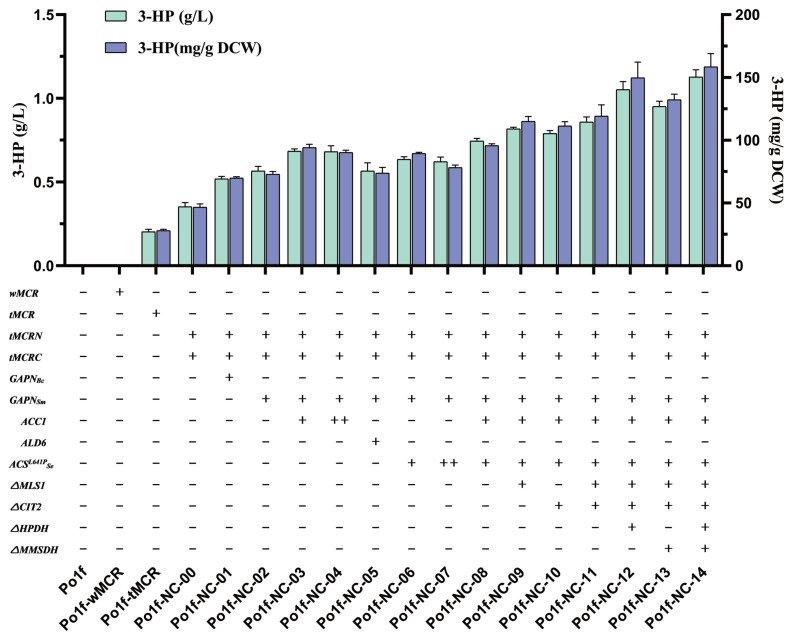
Quantitative analysis of 3-HP production in engineered *Y. lipolytica*. 3-HP productions in the engineered strains Po1f, Po1f-wMCR, Po1f-tMCR, Po1f-NC-00, Po1f-NC-01, Po1f-NC-02, Po1f-NC-03, Po1f-NC-04, Po1f-NC-05, Po1f-NC-06, Po1f-NC-07, Po1f-NC-08, Po1f-NC-09, Po1f-NC-10, Po1f-NC-11, Po1f-NC-12, Po1f-NC-13, and Po1f-NC-14. Three independent repeated experiments were performed for each strain. Error bars represent standard deviations.

As shown in Figure 1, generating 1 mole of 3-HP requires 2 moles of NADPH, while MCR${}_{Ca}$ requires more NADPH to synthesize higher yields of 3-HP. Therefore, it is necessary to improve the system’s reducing power. According to the literature, we introduced the non-phosphorylating glyceraldehyde-3-phosphate dehydrogenase gene (*GAPN*) from *B. cereus* and *S. mutans* into the MCR pathway to improve the NADPH level [37,38]. In order to supply NADPH to a greater extent, *GAPN${}_{Bc}$* and *GAPN${}_{Sm}$* were connected to the pINA1312 plasmid for overexpression, enabling more copies of the gene expression cassette to be integrated into the genome of *Y. lipolytica* through the NHEJ-mediated method. The gene expression cassettes of *GAPN${}_{Bc}$* and *GAPN${}_{Sm}$* were connected to p1312-tMCRN-tMCRC using a seamless connection method to obtain Plasmids p1312-NC-GAPN${}_{Bc}$ and p1312-NC-GAPN${}_{Sm}$. They were transformed to *Y. lipolytica*, and the yield of 3-HP of their fermentation broth was determined. According to the results (Figure 3), we found that supplying NADPH to the system can significantly increase 3-HP production. The yields of Po1f-NC-01 and Po1f-NC-02 fermentation broth reached 0.519 g·L^−1^ (DCW: 69.87 mg·g^−1^) and 0.566 g·L^−1^ (DCW: 72.83 mg·g^−1^), respectively. Therefore, *GAPN* from *S. mutans* is more suitable for expression in *Y. lipolytica*. In order to achieve higher yields, we chose strain Po1f-NC-02 for further modification. The map of plasmid p1312-NC-GAPN${}_{Sm}$ is shown in Figure S2.

### 3.3. Increasing Flux to 3-HP Production through Overexpression of MCR Pathway Genes

As shown in Figure 1, we attempted to enhance the metabolic flux from acetyl-CoA to malonyl-CoA using the overexpressed acetyl-CoA carboxylase 1 coding gene *ACC1* from *Y. lipolytica*, which was considered to have strong enzyme activity [20]. *ACC1${}_{Yl}$*, which was amplified from the genome of *Y. lipolytica*, was connected between the hp4d promoter and XPR2 terminator in plasmid pINA1269. The pINA1269 plasmid is helpful when studying the effect of the gene copy number on 3-HP production, as it can strictly control the number of copies of genes transformed into the genome. Plasmid p1269-ACC1 was transformed into the strain Po1f-NC-02 and integrated into the genome, obtaining strain Po1f-NC-03. Through quantitative HPLC analysis of the fermentation broth of strain Po1f-NC-03, we found that the concentration of 3-HP increased to 0.684 g·L^−1^ (DCW: 94.07 mg·g^−1^). As shown, an additional copy of *ACC1* can enhance the metabolic flux, thereby increasing 3-HP production. We attempted to connect another *ACC1* gene expression cassette based on p1269-ACC1, obtaining p1269-ACC1-ACC1. It was transformed into Po1f-NC-02, obtaining Po1f-NC-04 to further promote metabolic flux. Relative real-time quantitative analysis was performed on strains Po1f, Po1f-NC-03 and Po1f-NC-04 to confirm the effectiveness of gene manipulation. The ACC1 relative expression level is shown in Figure S1A. The results in Figure 3 show that the concentration of 3-HP in the fermentation broth of Po1f-NC-04 was 0.682 g·L^−1^ (DCW: 90.25 mg·g^−1^). The additional two copies did not lead to a higher 3-HP yield. It is inferred that *ACC1* is not the key limiting factor under this condition.

Meanwhile, the mutant *ACS${}^{L641P}$* gene from *Salmonella enterica* was overexpressed to provide the system with more acetyl-CoA [39]. *ACS${}_{Se}^{L641P}$* was connected to plasmid pINA1269 and transformed into strain Po1f-NC-02, obtaining Po1f-NC-06. The results showed that the concentration of 3-HP in fermentation broth of Po1f-NC-07 reached 0.635 g·L^−1^ (DCW: 89.4 mg·g^−1^). Therefore, the overexpression of *ACS${}_{Se}^{L641P}$* was effective in increasing 3-HP production. We attempted to connect another *ACS${}_{Se}^{L641P}$* gene expression cassette based on p1269-ACS${}_{Se}^{L641P}$, obtaining p1269-ACS${}_{Se}^{L641P}$-ACS${}_{Se}^{L641P}$. It was transformed into Po1f-NC-02, obtaining Po1f-NC-07. Relative real-time quantitative analysis was performed on strains Po1f-NC-06 and Po1f-NC-07 to confirm the expression level of ACS${}_{Se}^{L641P}$ enzyme. The ACS${}_{Se}^{L641P}$ relative expression level is shown in Figure S1B. The accumulation of 3-HP reached 0.622 g·L^−1^ (DCW: 78.16 mg·g^−1^), which is lower than that of Po1f-NC-06. This indicates that two copies of *ACS${}_{Se}^{L641P}$* affected the efficiency of gene expression, thereby having no effect on improving 3-HP production.

On this basis, we also explored the role of the overexpression of *ALD6* in improving 3-HP production. *ALD6${}_{Yl}$* was amplified from the genome of *Y. lipolytica* and connected to plasmid pINA1269, obtaining p1269-ALD6. Po1f-NC-05 was constructed by transforming p1269-ALD6 into the genome on the base of Po1f-NC-02. The yield of 3-HP reached 0.566 g·L^−1^ (DCW: 73.77 mg·g^−1^). This indicate that the overexpression of *ALD6${}_{Yl}$* cannot increase the metabolic flux to acetate compared to Po1f-NC-02. It can be inferred that *Y. lipolytica* does not proceed with aerobic alcohol fermentation, so the flux naturally flows to acetate [40]. This is one of the reasons why *Y. lipolytica* is more suitable for synthesizing 3-HP than *S. cerevisiae*.

Therefore, we finally chose to connect an *ACC1${}_{Yl}$* gene expression cassette and *ACS${}_{Se}^{L641P}$* gene expression cassette to plasmid pINA1269, obtaining p1269-ACC1-ACS${}_{Se}^{L641P}$. The map of plasmid ACS${}_{Se}^{L641P}$ is shown in Figure S3. The recombinant plasmid was transformed into the genome of Po1f-NC-02 to obtain Po1f-NC-08. The concentration of 3-HP in the fermentation broth of Po1f-NC-08 reached 0.745 g·L^−1^ (DCW: 95.72 mg·g^−1^), which was improved by 1.32-fold compared to that of Po1f-NC-02.

### 3.4. Knockout of Bypass Genes of Malonyl-CoA Pathway to Glyoxylate Cycle

To further improve 3-HP production and reduce metabolic flux to the bypass, we attempted to knock out the malate synthase 1 coding gene *MLS1* (YALI0D19140p) and citrate synthase 2 coding gene *CIT2* (YALI0E00638p), resulting in flux in the glyoxylate cycle. The CRISPR/Cas9 knockout system is suitable for the continuous knockout of multiple genes. We constructed plasmids JME4580-MLS1-sgRNA and JME4580-CIT2-sgRNA, which were connected by the corresponding sgRNA sequence. Meanwhile, the plasmids donor-MLS1 and donor-CIT2 were constructed using a seamless connection method which can provide donor fragments. Genes *MLS1* and *CIT2* were knocked out by the CRISPR/Cas9 system based on Po1f-NC-08. After DNA electrophoresis and Sanger sequencing verification, we obtained *MLS1* deletion transformant Po1f-NC-09 and *CIT2* deletion transformant Po1f-NC-10, with 3-HP yields of 0.818 g·L^−1^ (DCW: 115.05 mg·g^−1^) and 0.791 g·L^−1^ (DCW: 111.4 mg·g^−1^), respectively. It is indicated that the deletion of the two genes can reduce metabolic flux to the glyoxylate cycle, thereby increasing 3-HP production, which is consistent with the fermentation of 3-HP by *S. cerevisiae* [39]. Therefore, after culturing strain Po1f-NC-09 for 24 h to complete CRISPR/Cas9 plasmid curing, we knocked out *CIT2* on the basis of the strain Po1f-NC-09, finally obtaining Po1f-NC-11. The concentration of 3-HP reached 0.858 g·L^−1^ (DCW: 119.13 mg·g^−1^), an increase of 0.113 g·L^−1^ compared to that of the strain Po1f-NC-08.

### 3.5. Knockout of 3-HP Degradation Related Genes in Malonate Semialdehyde Pathway

**Figure 4:**
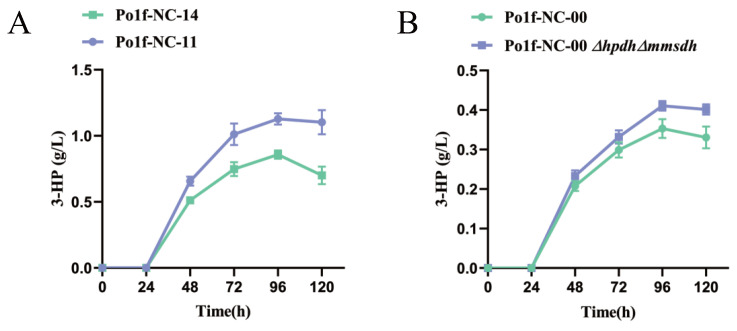
(**A**) 3-HP accumulation of strains Po1f-NC-11 and Po1f-NC-14 during 120 h fermentation. (**B**) 3-HP accumulation of strains Po1f-NC-00 and Po1f-NC-00 Δ*hpdh*Δ*mmsdh* during 120 h fermentation. Data represent the mean ± SD of biological triplicates in (**A**,**B**).

In order to further improve the concentration of 3-HP, we attempted to eliminate other factors that reduce its production. According to the results of Figure 2D, we found that the 3-HP yield of the strain Po1f-NC-00 decreased between 96 h and 120 h during fermentation. Considering that the characteristics of 3-HP are relatively stable under normal conditions, it is inferred that there is a 3-HP degradation pathway in *Y. lipolytica*. By querying the schematic diagram of the propanoate metabolism of *Y. lipolytica* in the KEGG database, the malonate semialdehyde pathway (M00013) from propanoyl-CoA to acetyl-CoA was found. As shown in Figure 1B, the 3-hydroxypropionate dehydrogenase coding gene *HPDH* (YALI0F02607p) can degrade 3-HP to malonate semialdehyde, and the methylmalonate-semialdehyde dehydrogenase coding gene *MMSDH* (YALI0C01859p) can degrade malonate semialdehyde to acetyl-CoA. Therefore, we attempted to knock out *HPDH* based on strain Po1f-NC-11, obtaining strain Po1f-NC-12. The concentration of 3-HP reached 1.053 g·L^−1^ (DCW: 149.74 mg·g^−1^), indicating that the deletion of *HPDH* can effectively alleviate 3-HP degradation. Since malonate semialdehyde is also an important intermediate product for converting malonyl-CoA to 3-HP, *MMSDH* was knocked out to increase the concentration of malonate semialdehyde. Strain Po1f-NC-13 was obtained by knocking out *MMSDH* based on the strain Po1f-NC-11. Its yield of 3-HP reached 0.952 g·L^−1^ (DCW: 132.24 mg·g^−1^). Therefore, preventing the degradation of malonate semialdehyde is beneficial for increasing the production of 3-HP. Finally, strain Po1f-NC-14 was obtained by knocking out *MMSDH* based on strain Po1f-NC-12. The concentration of the fermentation broth was 1.128 g·L^−1^ (DCW: 158.47 mg·g^−1^). To further verify the effect of the knockout malonate semialdehyde degradation pathway, we compared the concentration of 3-HP in the fermentation broth during 0–120 h of strains Po1f-NC-11 and Po1f-NC-14. It is shown in Figure 4A that as 3-HP synthesis began, knocking out genes *HPDH* and *MMSDH* increased 3-HP production, largely solving the degradation problem of 3-HP. Meanwhile, we knocked out genes *HPDH* and *MMSDH* based on the initial recombinant strain Po1f-NC-00, and the modification effect was consistent with that in Figure 4B. All of the aforementioned genetic operations improved the 3-HP titer by 5.55-fold in the fermentation broth of strain Po1f-NC-14 compared to that of Po1f-tMCR.

### 3.6. Effect of Pyruvic Acid Addition

**Figure 5:**
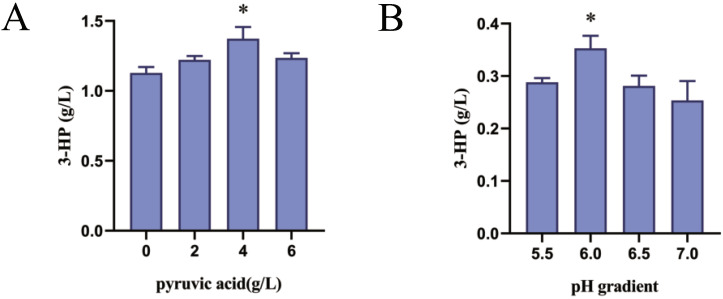

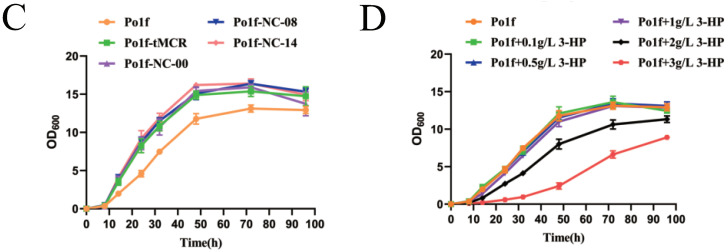
(**A**) Effects of 0 g·L^−1^, 2 g·L^−1^, 4 g·L^−1^ and 6 g·L^−1^ pyruvic acid added to YPD medium on the production of 3-HP in strain Po1f-NC-14. (**B**) Effects of YPD medium with pH values of 5.5, 6.0, 6.5, and 7.0 on the production of 3-HP in strain Po1f-NC-00. (**C**) The OD${}_{600}$ values of strains Po1f, Po1f-tMCR, Po1f-NC-00, Po1f-NC-08 and Po1f-NC-14 cultured in YPD medium, measured at 0, 8, 14, 24, 32, 48, 72, and 96 h. (**D**) The OD${}_{600}$ values of strains Po1f, Po1f adding 0.1 g·L^−1^ 3-HP, Po1f adding 0.5 g·L^−1^ 3-HP, Po1f adding 1 g·L^−1^ 3-HP, Po1f adding 2 g·L^−1^ 3-HP and Po1f adding 3 g·L^−1^ 3-HP cultured in YPD medium, measured at 0, 8, 14, 24, 32, 48, 72 and 96 h. Data represent the mean ± SD of biological triplicates in (**A**–**D**). Statistical analysis was conducted as significance indicated by * *p* < 0.05.

In addition to the modification of genes related to the malonyl-CoA pathway, changes in fermentation conditions can also increase 3-HP production. One option is the optimization of the fermentation medium. According to Figure 1, we attempted to add pyruvic acid, which was treated as an auxiliary carbon source for the medium under the premise of no glucose being added. Meanwhile, adding pyruvate acid can also push the metabolic flux towards acetic acid, increasing the production of 3-HP [41]. To explore the optimal concentration of pyruvate acid required to increase the yield of 3-HP, varying amounts of 0 to 6 g·L^−1^ pyruvate acid were added to the YPD medium, and their 3-HP production was prepared after culturing with a shake flask. As demonstrated by the results in Figure 5A, adding 4 g·L^−1^ pyruvate acid favored the 3-HP production best; the 3-HP yield of strain Po1f-NC-14 reached 1.373 g·L^−1^. Therefore, improving the yield of 3-HP can be attempted by adding pyruvate acid to the initial YPD medium, although this will increase the fermentation cost.

### 3.7. Fermentation-Relevant Characteristics of 3-HP Synthesis by Y. lipolytica

The pH of fermentation medium also affects the 3-HP yield. The initial pH value in this study was 6.0, and the previous experimental results were obtained under this condition. In order to find the optimal pH value and prepare for fermentation conditions optimization and bioreactor fermentation, we adjusted the pH of the YPD medium to 5.5, 6.0, 6.5, and 7.0, and performed fermentation experiments using strain Po1f-NC-00. The results shown in Figure 5B indicate that a pH of 6.0 is most suitable for the accumulation of 3-HP under current conditions, which may also depend on the acidity of the fermentation environment of *Y. lipolytica*; additionally, the strain was usually used to synthesize organic acids. However, the results also indicate that a pH lower than 6.0 is not beneficial for the accumulation of 3-HP. Therefore, we will continue to choose pH 6.0 for subsequent experiments.

The growth of initial strain Po1f and recombinant engineering strains should also be investigated. The growth of yeast is closely related to the production of 3-HP. The OD${}_{600}$ values of the strains Po1f, Po1f-tMCR, Po1f-NC-00, Po1f-NC-07 and Po1f-NC-14 cultured in YPD medium were determined at 0, 8, 14, 24, 32, 48, 72, and 96 h. The results in Figure 5C indicate that there was no significant difference in the growth of recombinant engineering, which was slightly faster than that of initial strain Po1f. It can be inferred that supplementing auxotroph markers to the genome of *Y. lipolytica* contributes to yeast growth. During shake flask fermentation, there is a good linearity correlation between OD${}_{600}$ and DCW.

On this basis, we explored the inhibitory effects of 3-HP on *Y. lipolytica*. Different concentrations of the 3-HP standard sample, which included 0.1 g·L^−1^, 0.5 g·L^−1^, 1 g·L^−1^, 2 g·L^−1^ and 3 g·L^−1^, were separately added to the YPD medium, and we used strain Po1f to perform fermentation experiments. The results in Figure 5D indicate that adding no more than 1 g·L^−1^ of the 3-HP standard sample to the YPD medium cannot affect the growth of strain Po1f. When the concentration of 3-HP added was greater than 2 g·L^−1^, yeast growth was affected. When the concentration of 3-HP added was greater than 3 g·L^−1^, the logarithmic growth phase of *Y. lipolytica* was delayed. In summary, it can be inferred that the accumulation of 3-HP during shake flask fermentation does not affect the normal growth of *Y. lipolytica* based on the current concentration of 3-HP in the fermentation broth of strain Po1f-NC-14.

### 3.8. Fed-Batch Fermentation of 3-HP on Strain Po1f-NC-14

**Figure 6:**
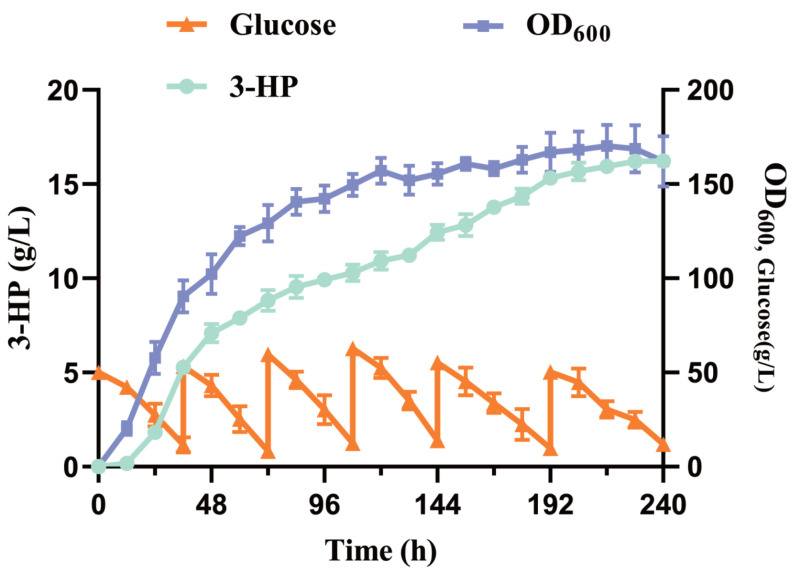
OD${}_{600}$ of growth, 3-HP accumulation and glucose consumption of strain Po1f-NC-14 in fed-batch fermentation in 1 L bioreactor. Data represent the mean ± SD of biological triplicates.

In order to further validate the synthesis effect of recombinant strain Po1f-NC-14 on 3-HP and explore the yield potential of fermentation preliminary, we performed fed-batch fermentation on strain Po1f-NC-14 in a 1 L bioreactor. The results in Figure 2D indicate that the synthesis of 3-HP stopped as glucose was depleted. Therefore, the initial concentration of glucose was raised to 50 g·L^−1^ to increase the production of 3-HP. Meanwhile, to avoid affecting the synthesis of 3-HP, we replenished glucose to the bioreactor to reach the initial concentration when the concentration of glucose was below 10 g·L^−1^. Suitable fermentation conditions for production 3-HP were set as follows: pH, 6.0; air flux, 1.5 vvm; and stirring rate, 550 rpm. As shown in Figure 6, the accumulation of 3-HP reached 16.23 g·L^−1^ at 240 h, which is the highest reported 3-HP yield in *Y. lipolytica*. The OD${}_{600}$ reached 170.2 at 216 h. The phenomenon of the OD value decreasing in the last 12 h of fermentation indicates that the strain began to die. At the same time, the concentration of 3-HP no longer increased after 228 h, indicating that the accumulation of the product was restricted by the growth of the strain and the fermentation conditions. The consumption rate of glucose gradually slowed in the later stage of fermentation, verifying that the viable count decreased. The optimization of fermentation conditions can further increase the expression level of relevant genes. Therefore, we could attempt to optimize bioreactor fermentation conditions in the future to pursue a higher yield.

## 4. Discussion

3-HP plays an important role in industrial chemical production. Chemical synthesis methods have a series of disadvantages, including high cost, high pollutant emissions, and complex steps, and utilizing microorganisms to synthesize 3-HP is environmentally friendly. The increasing yield of the biosynthesis method is also advantageous. Prokaryotic chassis cells, such as *E. coli* and *K. pneumoniae*, and eukaryotic chassis cells, such as *S. cerevisiae* and *P. pastoris*, were used to produce 3-HP. *E.coli*, which was previously used for research, as the chassis cell has the advantages of high yield and easy operation. However, when synthesizing 3-HP, it has poor tolerance, which is a disadvantage. *Y. lipolytica* has a high tolerance for the production of organic acid products because its fermentation environment is acidic. Compared to *S. cerevisiae*, *Y. lipolytica* also has more metabolic flux for synthesizing 3-HP. As malonyl-CoA is the precursor for the synthesis of FFA [42], *Y. lipolytica*, a strain capable of synthesizing and storing large amounts of FFA, has a significant advantage in producing 3-HP . Therefore, we chose *Y. lipolytica* as the chassis cell to synthesize 3-HP in this study.

As shown in Section 3.1, the initial malonyl-CoA pathway for synthesizing 3-HP was constructed in *Y. lipolytica*. The yield of 3-HP reached 0.203 g·L^−1^, which is higher than that of *S. cerevisiae* [17]. This also indicates that *Y. lipolytica* has a more abundant precursor than *S. cerevisiae*. In order to further enhance enzyme activity, according to the literature, MCR was split into two subunits: MCR-N and MCR-C. The yield of 3-HP reached 0.353 g·L^−1^. The activity of MCR and replenishing of NADPH are the key to increasing the yield of 3-HP through the malonyl-CoA pathway [43]. Therefore, plasmid pINA1312 was used to connect their coding genes to perform multiple copies into genome of *Y. lipolytica* by NHEJ [44]. The results indicated that *GAPN${}_{Sm}$* is more suitable for expression than *GAPN${}_{Bc}$* as described in Section 3.2. On this basis, a series of genes was overexpressed to promote the metabolic flux to flow towards malonyl-CoA. As shown in Section 3.3, one overexpressed copy of *ACC1${}_{Yl}$* and *ACS${}_{Se}^{L641P}$* by plasmid pINA1269 can increase the yield of 3-HP to 0.745 g·L^−1^. The results showed that transforming another two copies of the two genes into the genome of *Y. lipolytica* failed to improve the yield of 3-HP. It is inferred that the contents of acetate and acetyl-CoA in the strain are not the key factor limiting 3-HP production at this time. Likewise, the overexpression of the *ALD6* gene cannot increase the production of 3-HP; this may be because *Y. lipolytica* is unable to perform aerobic alcohol fermentation, and the main metabolic flux automatically flows to acetate. This is not consistent with the situation in *S. cerevisiae*. In Section 3.4, we used the CRISPR/Cas9 system to knock out genes related to the glyoxylate cycle, including *MLS1* and *CIT2*. The yield of 3-HP increased by 0.113 g·L^−1^, which is higher than that of *S. cerevisiae*. By focusing on the production of 3-HP in the later stage of fermentation, we noticed the degradation of 3-HP. After querying the KEGG database, we discovered the presence of the malonate semialdehyde pathway, which can degrade 3-HP in *Y. lipolytica*. In Section 3.5, the genes *MMSDH* and *HPDH* were knocked out to alleviate the degradation of 3-HP and increase the content of precursor for 3-HP synthesis. The yield of strain reached 1.128 g·L^−1^, increasing by 0.269 g·L^−1^, and significantly improved the production. Compared to the initial strain Po1f-tMCR, the yield of 3-HP improved 5.55-fold. This yield is higher than that of the recombinant *S. cerevisiae* strain achieved via shake flask culturing [45]. In Section 3.6 and Section 3.7, a series of fermentation-level measures were implemented to increase the production of 3-HP. Adding 4 g·L^−1^ pyruvic acid to the YPD medium can further increase the production of 3-HP because pyruvic acid is also another carbon source. In the background of shake flask fermentation culture, the medium with a pH value of 6.0 is more suitable for the accumulation of 3-HP. OD${}_{600}$ is a reflection of the growth status of the strain. The results showed that there is no significant difference in the OD${}_{600}$ among different recombinant strains. This situation is consistent with the growth of recombinant strains of *Y. lipolytica*, which are used to produce other productions. To rule out the impact of 3-HP accumulation on strain growth, we tested the tolerance of *Y. lipolytica* to 3-HP. The results showed that the current 3-HP shake flask fermentation yield does not impact the growth of *Y. lipolytica*. Lastly, fed-batch fermentation was performed in a 1 L bioreactor to preliminarily verify the ability of strain Po1f-NC-14 to synthesize 3-HP in Section 3.8. The accumulation concentration of 3-HP in the fermentation broth of Po1f-NC-14 reached 16.23 g·L^−1^, which is higher than that in *E. coli* [11] and is close to that of an unconventional yeast *P. pastoris* [20]. Through the fed-batch fermentation results, it can also be observed that the OD${}_{600}$ began to decrease and the speed rate of glucose consumption gradually slowed. As a result, the production of 3-HP no longer increased in the later stage of fermentation. It can be inferred that the volume of bioreactor is an important factor which limits strain growth and the yield of 3-HP.

Although we employed a series of measures to increase the production of 3-HP, there are still many new directions to explore. Constructing a super yeast chassis can significantly improve the metabolic flux of precursor for synthesizing 3-HP, and related gene manipulations can be applied in *Y. lipolytica* [42]. We will also perform gene manipulation on malate dehydrogenase coding gene (*MDH*) and focus on the ration of two forms of MDH to further increase the yield of 3-HP. Attempting to apply differ expression promoters [46], setting up a dynamic sensor–regulator system to control the expression level of genes [47], and overexpressing relevant genes obtained from the results of RNA-sequencing of recombinant strain [48] can increase 3-HP production at the genetic level. At the same time, optimizing the fermentation conditions of fed-batch fermentation in the bioreactor can also improve the accumulation of 3-HP. Overall, using *Y. lipolytica* to produce and accumulate 3-HP has great potential for development.

## 5. Conclusions

In this paper, we obtained the recombinant *Y. lipolytica* Po1f-NC-14 by overexpressed genes *MCR-N${}_{Ca}$*, *MCR-C${}_{Ca}$*, *GAPN${}_{Sm}$*, *ACC1* and *ACS${}_{Se}^{L641P}$*, knocking out genes *MLS1* and *CIT2*, leading to the glyoxylate cycle, and knocking out 3-HP degradation-related genes *MMSDH* and *HPDH*. The yield of 3-HP in shake flask fermentation reached 1.128 g·L^−1^, and the yield in a fed-batch fermentation in a 1 L bioreactor reached 16.23 g·L^−1^. We also analyzed the impact of changing fermentation conditions on 3-HP production and the tolerance of *Y. lipolytica* to the product. This study utilized *Y. lipolytica* to produce 3-HP, creating new directions of research and providing insights into the synthesis of organic acid in unconventional yeast, which will provide a reference for future studies.

## Figures and Tables

**Table 1:** Plasmids used in this study.

Plasmid	Description	Reference
pINA1312	*Y. lipolytica*-integrative plasmid, hp4d promoter, XPR2 terminator, ura3d1 selection marker, Km${}^{R}$	[26]
pINA1269	*Y. lipolytica*-integrative plasmid, hp4d promoter, XPR2 terminator, LEU2 selection marker, Amp${}^{R}$	[26]
pTrc99a-wMCR	pTrc99a plasmid contains wild *MCR* gene from *C. aurantiacus*	[12]
p1312-wMCR	pINA1312 plasmid contains wild *MCR* gene from *C. aurantiacus*	this study
p1312-tMCR	pINA1312 plasmid contains codon-optimized *MCR* gene from *C. aurantiacus*	this study
p1312-tMCRN-tMCRC	pINA1312 plasmid contains codon-optimized *MCR-N* gene cassette and *MCR-C* gene cassette	this study
p1312-NC-*GAPN${}_{Bc}$*	pINA1312 plasmid contains codon-optimized *MCR-N/C* gene cassettes and codon optimized *GAPN* gene cassette from *Bacillus cereus*	this study
p1312-NC-*GAPN${}_{Sm}$*	pINA1312 plasmid contains codon-optimized *MCR-N/C* gene cassettes and codon optimized *GAPN* gene cassette from *Streptococcus mutans*	this study
p1269-ACC1	pINA1269 plasmid contains *ACC1* gene from *Y. lipolytica*	this study
p1269-ACC1-ACC1	pINA1269 plasmid contains two *ACC1* gene cassettes	this study
p1269-*ACS${}_{Se}^{L641P}$*	pINA1269 plasmid contains codon-optimized mutant *ACS${}^{L641P}$* gene from *Salmonella enterica*	this study
p1269-*ACS${}_{Se}^{L641P}$*-*ACS${}_{Se}^{L641P}$*	pINA1269 plasmid contains two *ACS${}_{Se}^{L641P}$* gene cassettes	this study
p1269-ALD6	pINA1269 plasmid contains *ALD6* gene from *Y. lipolytica*	this study
p1269-ACC1-*ACS${}_{Se}^{L641P}$*	pINA1269 plasmid contains *ACC1* gene cassette and mutant *ACS${}_{Se}^{L641P}$* gene cassette	this study
JME4580	*Y. lipolytica* CRISPR/Cas9 plasmid, HPHex marker for sgRNA cloning, RFP, Amp${}^{R}$	Addgene
JME4580-MLS1-sgRNA	JME4580 plasmid contains sgRNA of *MLS1* gene instead of RFP expression gene	this study
JME4580-CIT2-sgRNA	JME4580 plasmid contains sgRNA of *CIT2* gene instead of RFP expression gene	this study
JME4580-HPDH-sgRNA	ME4580 plasmid contains sgRNA of *HPDH* gene instead of RFP expression gene	this study
JME4580-MMSDH-sgRNA	JME4580 plasmid contains sgRNA of *MMSDH* gene instead of RFP expression gene	this study
pCE-Zero	For constructing CRISPR/Cas9 donor fragment, Amp${}^{R}$, Km${}^{R}$, linearized	Vazyme
donor-MLS1	pCE-Zero plasmid contains donor fragment of 1kb upstream and 1kb downstream of *MLS1* gene (YALI0D19140g)	this study
donor-CIT2	pCE-Zero plasmid contains donor fragment of 1kb upstream and 1kb downstream of *CIT2* gene (YALI0E00638g)	this study
donor-HPDH	pCE-Zero plasmid contains donor fragment of 1kb upstream and 1kb downstream of *HPDH* gene (YALI0F02607g)	this study
donor-MMSDH	pCE-Zero plasmid contains donor fragment of 1kb upstream and 1kb downstream of *MMSDH* gene (YALI0C01859g)	this study

## Data Availability

The data presented in this study are available on request from the corresponding author.

## Supplementary Materials

The following supporting information can be downloaded at: https://www.mdpi.com/article/10.3390/jof9050573/s1, Table S1: Sequences of genes investigated in this study; Table S2: sgRNAs used in this study; Figure S1: (A) The relative ACC1 expression of strain Po1f, Po1f-NC-03 and Po1f-NC-04; (B) The relative *ACS${}_{Se}^{L641P}$* expression of strain Po1f, Po1f-NC-06 and Po1f-NC-07; Figure S2: The map of plasmid p1312-NC-GAPN${}_{Sm}$; Figure S3: The map of plasmid p1269-ACC1-*ACS${}_{Se}^{L641P}$*.
