# Supplementary material for: Engineering 3-Hydroxypropionic Acid Production from Glucose in Yarrowia lipolytica through Malonyl-CoA Pathway
Source: J Fungi (Basel). 2023 May 15;9(5):573. doi: 10.3390/jof9050573 (PMC10219321; doi:10.3390/jof9050573)
Supplement: Supplementary file 1 [file jof-09-00573-s001.zip › jof-2379335-supplementary.pdf]

Supplementary Materials

Engineering 3-Hydroxypropionic Acid Production from Glucose in *Yarrowia lipolytica* though Malonyl-CoA Pathway

Table S1. Sequences of genes investigated in this study.

Gene	Sequence
MCR$_{Ca}$<br>(GenBank:<br>CP000909.1)	*ATGAGCGGAACAGGACGACTGGCAGGAAAGATTGCGTTAATTACCGGTGGCGCCGGCAATATCGGCAGTGAATTGACACGTCGCTTTCTCGCAGAG<br>GGAGCGACGGTCATTATTAGTGGACGGAATCGGGCGAAGTTGACCGCACTGGCCGAACGGATGCAGGCAGAGGCAGGAGTGCCGGCAAAGCGCA<br>TCGATCTCGAAGTCATGGATGGGAGTGATCCGGTCGCGGTACGTGCCGGTATCGAAGCGATTGTGGCCCGTCACGGCCAGATCGACATTCTGGTC<br>AACAATGCAGGAAGTGCCGGTGCCCAGCGTCGTCTGGCCGAGATTCCACTCACTGAAGCTGAATTAGGCCCTGGCGCCGAAGAGACGCTTCATGC<br>CAGCATCGCCAATTTACTTGGTATGGGATGGCATCTGATGCGTATTGCGGCACCTCATATGCCGGTAGGAAGTGCGGTCATCAATGTCTCGACCATC<br>TTTTCACGGGCTGAGTACTACGGGCGGATTCCGTATGTCACCCCTAAAGCTGCTCTTAATGCTCTATCTCAACTTGCTGCGCGTGAGTTAGGTGCAC<br>GTGGCATCCGCGTTAATACGATCTTTCCCGGCCCGATTGAAAGTGATCGCATCCGTACAGTGTTCCAGCGTATGGATCAGCTCAAGGGGCGGCCCG<br>AAGGCGACACAGCGCACCATTTTTTGAACACCATGCGATTGTGTCGTGCCAACGACCAGGGCGCGCTTGAACGTCGGTTCCCCTCCGTCGGTGATG<br>TGGCAGACGCCGCTGTCTTTCTGGCCAGTGCCGAATCCGCCGCTCTCTCCGGTGAGACGATTGAGGTTACGCACGGAATGGAGTTGCCGGCCTGC<br>AGTGAGACCAGCCTGCTGGCCCGTACTGATCTGCGCACGATTGATGCCAGTGGCCGCACGACGCTCATCTGCGCCGGCGACCAGATTGAAGAGGT<br>GATGGCGCTCACCGGTATGTTGCGTACCTGTGGGAGTGAAGTGATCATCGGCTTCCGTTCGGCTGCGGCGCTGGCCCAGTTCGAGCAGGCAGTCA<br>ATGAGAGTCGGCGGCTGGCCGGCGCAGACTTTACGCCTCCCATTGCCTTGCCACTCGATCCACGCGATCCGGCAACAATTGACGCTGTCTTCGATT<br>GGGCCGGCGAGAATACCGGCGGGATTCATGCAGCGGTGATTCTGCCTGCTACCAGTCACGAACCGGCACCGTGCGTGATTGAGGTTGATGATGAG<br>CGGGTGCTGAATTTTCTGGCCGATGAAATCACCGGGACAATTGTGATTGCCAGTCGCCTGGCCCGTTACTGGCAGTCGCAACGGCTTACCCCCGGC<br>GCACGTGCGCGTGGGCCGCGTGTCATTTTTCTCTCGAACGGTGCCGATCAAAATGGGAATGTTTACGGACGCATTCAAAGTGCCGCTATCGGTCAG<br>CTCATTCGTGTGTGGCGTCACGAGGCTGAACTTGACTATCAGCGTGCCAGCGCCGCCGGTGATCATGTGCTGCCGCCGGTATGGGCCAATCAGATT<br>GTGCGCTTCGCTAACCGCAGCCTTGAAGGGTTAGAATTTGCCTGTGCCTGGACAGCTCAATTGCTCCATAGTCAACGCCATATCAATGAGATTACCC<br>TCAACATCCCTGCCAACATTAGCGCCACCACCGGCGCACGCAGTGCATCGGTCGGATGGGCGGAAAGCCTGATCGGGTTGCATTTGGGGAAAGTT<br>GCCTTGATTACCGGTGGCAGCGCCGGTATTGGTGGGCAGATCGGGCGCCTCCTGGCTTTGAGTGGCGCGCGCGTGATGCTGGCAGCCCGTGATC<br>GGCATAAGCTCGAACAGATGCAGGCGATGATCCAATCTGAGCTGGCTGAGGTGGGGTATACCGATGTCGAAGATCGCGTCCACATTGCACCGGGC<br>TGCGATGTGAGTAGCGAAGCGCAGCTTGCGGATCTTGTTGAACGTACCCTGTCAGCTTTTGGCACCGTCGATTATCTGATCAACAACGCCGGGATC<br>GCCGGTGTCGAAGAGATGGTTATCGATATGCCAGTTGAGGGATGGCGCCATACCCTCTTCGCCAATCTGATCAGCAACTACTCGTTGATGCGCAAAC<br>TGGCGCCGTTGATGAAAAAACAGGGTAGCGGTTACATCCTTAACGTCTCATCATACTTTGGCGGTGAAAAAGATGCGGCCATTCCCTACCCCAACCG<br>TGCCGATTACGCCGTCTCGAAGGCTGGTCAGCGGGCAATGGCCGAAGTCTTTGCGCGCTTCCTTGGCCCGGAGATACAGATCAATGCCATTGCGC<br>CGGGTCCGGTCGAAGGTGATCGCTTGCGCGGTACCGGTGAACGTCCCGGCCTCTTTGCCCGTCGGGCGCGGCTGATTTTGGAGAACAAGCGGCT<br>GAATGAGCTTCACGCTGCTCTTATCGCGGCTGCGCGCACCGATGAGCGATCTATGCACGAACTGGTTGAACTGCTCTTACCCAATGATGTGGCCGC<br>ACTAGAGCAGAATCCCGCAGCACCTACCGCGTTGCGTGAACTGGCACGACGTTTTCGCAGCGAAGGCGATCCGGCGGCATCATCAAGCAGTGCGC<br>TGCTGAACCGTTCAATTGCCGCTAAATTGCTGGCTCGTTTGCATAATGGTGGCTATGTGTTGCCTGCCGACATCTTTGCAAACCTGCCAAACCCGCC<br>CGATCCCTTCTTCACCCGAGCCCAGATTGATCGCGAGGCTCGCAAGGTTCGTGACGGCATCATGGGGATGCTCTACCTGCAACGGATGCCGACTGA<br>GTTTGATGTCGCAATGGCCACCGTCTATTACCTTGCCGACCGCAATGTCAGTGGTGAGACATTCCACCCATCAGGTGGTTTGCGTTACGAACGCACC<br>CCTACCGGTGGCGAACTCTTCGGCTTGCCCTCACCGGAACGGCTGGCGGAGCTGGTCGGAAGCACGGTCTATCTGATAGGTGAACATCTGACTGA<br>ACACCTTAACCTGCTTGCCCGTGCGTACCTCGAACGTTACGGGGCACGTCAGGTAGTGATGATTGTTGAGACAGAAACCGGGGCAGAGACAATGCG<br>TCGCTTGCTCCACGATCACGTCGAGGCTGGTCGGCTGATGACTATTGTGGCCGGTGATCAGATCGAAGCCGCTATCGACCAGGCTATCACTCGCTA<br>CGGTCGCCCAGGGCCGGTCGTCTGTACCCCCTTCCGGCCACTGCCGACGGTACCACTGGTCGGGCGTAAAGACAGTGACTGGAGCACAGTGTTGA<br>GTGAGGCTGAATTTGCCGAGTTGTGCGAACACCAGCTCACCCACCATTTCCGGGTAGCGCGCAAGATTGCCCTGAGTGATGGTGCCAGTCTCGCGC<br>TGGTCACTCCCGAAACTACGGCTACCTCAACTACCGAGCAATTTGCTCTGGCTAACTTCATCAAAACGACCCTTCACGCTTTTACGGCTACGATTGGT<br>GTCGAGAGCGAAAGAACTGCTCAGCGCATTCTGATCAATCAAGTCGATCTGACCCGGCGTGCGCGTGCCGAAGAGCCGCGTGATCCGCACGAGCG<br>TCAACAAGAACTGGAACGTTTTATCGAGGCAGTCTTGCTGGTCACTGCACCACTCCCGCCTGAAGCCGATACCCGTTACGCCGGGCGGATTCATCG<br>CGGACGGGCGATTACCGTGTAA*
Codon-optimized<br>*MCR*$_{Ca}$ gene<br>sequence	*ATGTCTGGCACCGGCCGACTGGCCGGCAAGATCGCCCTGATCACCGGCGGAGCCGGCAACATCGGCTCTGAGCTGACCCGACGATTCCTGGCCG<br>AGGGCGCCACCGTGATCATCTCTGGCCGAAACCGAGCCAAGCTGACCGCTCTGGCTGAGCGAATGCAGGCCGAGGCCGGCGTGCCCGCCAAGCG<br>AATCGACCTGGAAGTGATGGACGGCTCTGACCCCGTGGCCGTGCGAGCCGGCATCGAGGCCATCGTGGCCCGACACGGCCAGATCGACATCCTG<br>GTGAACAACGCCGGCTCTGCTGGCGCCCAGCGACGACTGGCTGAGATTCCCCTGACCGAGGCCGAGCTTGGACCCGGCGCTGAGGAAACCCTGC<br>ACGCCTCTATCGCCAACCTGCTCGGCATGGGCTGGCACCTGATGCGAATCGCCGCTCCTCACATGCCCGTGGGCTCTGCCGTGATCAACGTGTCTA<br>CCATCTTCTCGCGAGCCGAGTACTACGGACGAATCCCCTACGTGACCCCTAAGGCCGCTCTGAACGCCCTGTCTCAGCTGGCCGCTCGAGAGCTG<br>GGAGCCCGAGGCATCCGAGTGAACACCATCTTTCCCGGACCTATCGAGTCTGACCGAATCCGAACCGTGTTCCAGCGAATGGACCAGCTGAAGGG<br>ACGACCCGAGGGCGATACCGCTCACCACTTCCTCAACACCATGCGACTGTGCCGAGCCAACGACCAGGGTGCCCTCGAGCGACGATTTCCCTCTG<br>TGGGCGACGTGGCCGACGCCGCCGTGTTCCTGGCCTCTGCCGAGTCTGCCGCTCTGTCTGGCGAGACTATCGAGGTGACCCACGGCATGGAACT<br>GCCCGCCTGCTCTGAGACTTCCCTGCTGGCCCGAACCGACCTGCGAACCATCGACGCCTCTGGACGAACCACTCTGATCTGTGCCGGCGACCAGA<br>TCGAAGAGGTGATGGCCCTGACCGGCATGCTGCGAACCTGTGGCTCTGAGGTGATCATCGGCTTCCGATCTGCCGCCGCTCTCGCCCAGTTCGAG<br>CAGGCCGTGAACGAGTCCCGACGACTCGCTGGCGCCGACTTCACCCCTCCTATCGCTCTGCCCCTGGATCCTCGAGATCCCGCCACCATTGACGC<br>TGTGTTCGACTGGGGAGCCGGCGAGAACACCGGCGGCATCCACGCCGCTGTGATTCTGCCCGCCACCTCTCACGAGCCCGCACCTTGCGTGATCG<br>AGGTGGACGACGAGCGAGTGCTGAACTTCCTGGCTGACGAGATCACCGGAACCATCGTGATCGCCTCTCGACTGGCCCGATACTGGCAGTCTCAG*

	CGACTGACCCCTGGCGCTCGAGCACGAGGACCCCGAGTGATCTTCCTGTCTAACGGCGCCGACCAGAACGGCAACGTGTACGGCCGAATCCAGTC<br>*CGCCGCCATCGGACAGCTGATTCGAGTGTGGCGACACGAGGCTGAGCTGGACTACCAGCGAGCCTCCGCCGCTGGCGACCACGTGCTGCCTCCA*<br>*GTGTGGGCCAACCAGATCGTGCGATTCGCCAACCGATCTCTCGAGGGACTCGAGTTCGCCTGCGCCTGGACCGCTCAGCTGCTGCACTCCCAGCG*<br>*ACACATCAACGAGATTACCCTGAACATCCCCGCCAACATCTCTGCCACCACCGGCGCTCGATCTGCCTCTGTCGGCTGGGCCGAGTCTCTGATCGG*<br>*ACTGCACCTCGGCAAGGTGGCCCTCATTACCGGTGGATCTGCCGGAATCGGAGGACAGATCGGTCGACTGCTGGCTCTGTCCGGTGCTCGAGTGA*<br>*TGCTGGCTGCCCGAGATCGACACAAGCTCGAGCAGATGCAGGCCATGATCCAGTCTGAGCTGGCTGAGGTGGGCTACACCGACGTCGAGGACCGA*<br>*GTGCACATTGCTCCCGGCTGCGACGTGTCCTCTGAGGCCCAGCTCGCCGACCTGGTCGAGCGAACCCTGTCTGCCTTCGGCACCGTGGACTACCT*<br>*GATCAACAACGCTGGAATCGCCGGCGTGGAAGAGATGGTGATTGACATGCCTGTGGAAGGCTGGCGACACACCCTGTTCGCTAACCTGATCTCCAA*<br>*CTACTCTCTCATGCGAAAGCTGGCCCCTCTGATGAAGAAGCAAGGCTCTGGCTATATCCTGAACGTGTCGTCTTACTTCGGCGGCGAGAAGGACGC*<br>*CGCTATTCCCTATCCTAACCGAGCCGACTACGCCGTGTCTAAGGCCGGACAGCGAGCCATGGCCGAGGTGTTCGCCCGATTTCTGGGCCCCGAGA*<br>*TCCAGATCAACGCTATCGCCCCTGGACCTGTCGAGGGCGACCGACTGCGAGGCACCGGCGAGCGACCCGGCCTGTTTGCCCGACGAGCCCGACT*<br>*GATCCTGGAAAACAAGCGACTGAACGAGCTGCACGCTGCCCTGATCGCTGCCGCTCGAACCGATGAGCGATCTATGCACGAGCTGGTGGAACTGC*<br>*TGCTGCCCAACGACGTGGCTGCCCTGGAACAGAACCCCGCTGCTCCCACCGCTCTGCGAGAACTGGCCAGACGATTCCGATCCGAGGGTGACCCT*<br>*GCCGCTTCTTCCTCTTCTGCCCTGCTGAACCGATCTATCGCCGCCAAGCTCCTGGCTCGACTGCACAACGGCGGCTACGTGCTCCCCGCCGACATT*<br>*TTTGCTAACCTGCCTAACCCTCCTGATCCATTCTTTACTCGAGCCCAGATTGACCGAGAGGCCCGAAAGGTGCGAGATGGCATCATGGGCATGCTGT*<br>*ACCTGCAGCGAATGCCCACCGAGTTCGACGTCGCCATGGCCACCGTCTACTACCTGGCCGACCGAAACGTGTCCGGTGAGACTTTTCACCCTTCTG*<br>*GCGGCCTGCGATACGAGCGAACTCCCACCGGCGGTGAGCTGTTCGGACTGCCTTCGCCTGAGCGACTGGCAGAGCTGGTCGGCTCTACCGTGTA*<br>*CCTGATCGGCGAGCACCTCACCGAGCACCTGAACCTGCTTGCCCGAGCCTACCTTGAGCGATACGGTGCCCGACAGGTGGTGATGATCGTCGAGA*<br>*CTGAGACTGGTGCCGAGACTATGCGACGACTCCTGCACGACCACGTCGAGGCCGGACGACTGATGACCATTGTGGCTGGTGACCAGATTGAGGCC*<br>*GCCATTGACCAGGCTATCACCCGATACGGTCGACCCGGACCAGTGGTGTGCACCCCTTTCCGACCTCTGCCTACCGTGCCTCTGGTGGGCCGAAA*<br>*GGACTCTGACTGGTCTACCGTGCTGTCTGAGGCCGAGTTCGCTGAGCTGTGCGAGCACCAGCTGACCCACCACTTTCGAGTCGCCCGAAAGATTGC*<br>*CCTGTCCGACGGCGCCTCTCTGGCCCTGGTGACCCCTGAGACTACCGCTACCTCTACCACCGAGCAGTTCGCCCTGGCCAACTTCATCAAGACCAC*<br>*TCTGCACGCTTTCACCGCTACCATCGGCGTCGAGTCGGAGCGAACAGCCCAGCGAATCCTGATTAACCAGGTGGACCTGACTCGACGAGCTAGAG*<br>*CCGAGGAACCCAGAGATCCCCACGAGCGACAGCAAGAGCTGGAACGATTCATCGAGGCTGTGCTGCTGGTCACCGCTCCACTGCCACCTGAGGCT*<br>*GACACTCGATACGCCGGACGAATTCACCGAGGACGAGCCATCACCGTGTAA*
Codon-optimized MCR_{Ca}-*N* gene sequence	*ATGTCTGGCACCGGCCGACTGGCCGGCAAGATCGCCCTGATCACCGGCGGAGCCGGCAACATCGGCTCTGAGCTGACCCGACGATTCCTGGCCG*<br>*AGGGCGCCACCGTGATCATCTCTGGCCGAAACCGAGCCAAGCTGACCGCTCTGGCTGAGCGAATGCAGGCCGAGGCCGGCGTGCCCGCCAAGCG*<br>*AATCGACCTGGAAGTGATGGACGGCTCTGACCCCGTGGCCGTGCGAGCCGGCATCGAGGCCATCGTGGCCCGACACGGCCAGATCGACATCCTG*<br>*GTGAACAACGCCGGCTCTGCTGGCGCCCAGCGACGACTGGCTGAGATTCCCCTGACCGAGGCCGAGCTTGGACCCGGCGCTGAGGAAACCCTGC*<br>*ACGCCTCTATCGCCAACCTGCTCGGCATGGGCTGGCACCTGATGCGAATCGCCGCTCCTCACATGCCCGTGGGCTCTGCCGTGATCAACGTGTCTA*<br>*CCATCTTCTCGCGAGCCGAGTACTACGGACGAATCCCCTACGTGACCCCTAAGGCCGCTCTGAACGCCCTGTCTCAGCTGGCCGCTCGAGAGCTG*<br>*GGAGCCCGAGGCATCCGAGTGAACACCATCTTTCCCGGACCTATCGAGTCTGACCGAATCCGAACCGTGTTCCAGCGAATGGACCAGCTGAAGGG*<br>*ACGACCCGAGGGCGATACCGCTCACCACTTCCTCAACACCATGCGACTGTGCCGAGCCAACGACCAGGGTGCCCTCGAGCGACGATTTCCCTCTG*<br>*TGGGCGACGTGGCCGACGCCGCCGTGTTCCTGGCCTCTGCCGAGTCTGCCGCTCTGTCTGGCGAGACTATCGAGGTGACCCACGGCATGGAACT*<br>*GCCCGCCTGCTCTGAGACTTCCCTGCTGGCCCGAACCGACCTGCGAACCATCGACGCCTCTGGACGAACCACTCTGATCTGTGCCGGCGACCAGA*<br>*TCGAAGAGGTGATGGCCCTGACCGGCATGCTGCGAACCTGTGGCTCTGAGGTGATCATCGGCTTCCGATCTGCCGCCGCTCTCGCCCAGTTCGAG*<br>*CAGGCCGTGAACGAGTCCCGACGACTCGCTGGCGCCGACTTCACCCCTCCTATCGCTCTGCCCCTGGATCCTCGAGATCCCGCCACCATTGACGC*<br>*TGTGTTCGACTGGGGAGCCGGCGAGAACACCGGCGGCATCCACGCCGCTGTGATTCTGCCCGCCACCTCTCACGAGCCCGCACCTTGCGTGATCG*<br>*AGGTGGACGACGAGCGAGTGCTGAACTTCCTGGCTGACGAGATCACCGGAACCATCGTGATCGCCTCTCGACTGGCCCGATACTGGCAGTCTCAG*<br>*CGACTGACCCCTGGCGCTCGAGCACGAGGACCCCGAGTGATCTTCCTGTCTAACGGCGCCGACCAGAACGGCAACGTGTACGGCCGAATCCAGTC*<br>*CGCCGCCATCGGACAGCTGATTCGAGTGTGGCGACACGAGGCTGAGCTGGACTACCAGCGAGCCTCCGCCGCTGGCGACCACGTGCTGCCTCCA*<br>*GTGTGGGCCAACCAGATCGTGCGATTCGCCAACCGATCTCTCGAGGGACTCGAGTTCGCCTGCGCCTGGACCGCTCAGCTGCTGCACTCCCAGCG*<br>*ACACATCAACGAGATTACCCTGAACATCCCCGCCAACATCTAA*
Codon-optimized MCR_{Ca}-*C* gene sequence	*ATGTCTGCCACCACCGGCGCTCGATCTGCCTCTGTCGGCTGGGCCGAGTCTCTGATCGGACTGCACCTCGGCAAGGTGGCCCTCATTACCGGTGG*<br>*ATCTGCCGGAATCGGAGGACAGATCGGTCGACTGCTGGCTCTGTCCGGTGCTCGAGTGATGCTGGCTGCCCGAGATCGACACAAGCTCGAGCAGA*<br>*TGCAGGCCATGATCCAGTCTGAGCTGGCTGAGGTGGGCTACACCGACGTCGAGGACCGAGTGCACATTGCTCCCGGCTGCGACGTGTCCTCTGAG*<br>*GCCCAGCTCGCCGACCTGGTCGAGCGAACCCTGTCTGCCTTCGGCACCGTGGACTACCTGATCAACAACGCTGGAATCGCCGGCGTGGAAGAGAT*<br>*GGTGATTGACATGCCTGTGGAAGGCTGGCGACACACCCTGTTCGCTAACCTGATCTCCAACTACTCTCTCATGCGAAAGCTGGCCCCTCTGATGAAG*<br>*AAGCAAGGCTCTGGCTATATCCTGAACGTGTCGTCTTACTTCGGCGGCGAGAAGGACGCCGCTATTCCCTATCCTAACCGAGCCGACTACGCCGTG*<br>*TCTAAGGCCGGACAGCGAGCCATGGCCGAGGTGTTCGCCCGATTTCTGGGCCCCGAGATCCAGATCAACGCTATCGCCCCTGGACCTGTCGAGGG*<br>*CGACCGACTGCGAGGCACCGGCGAGCGACCCGGCCTGTTTGCCCGACGAGCCCGACTGATCCTGGAAAACAAGCGACTGAACGAGCTGCACGCT*<br>*GCCCTGATCGCTGCCGCTCGAACCGATGAGCGATCTATGCACGAGCTGGTGGAACTGCTGCTGCCCAACGACGTGGCTGCCCTGGAACAGAACCC*<br>*CGCTGCTCCCACCGCTCTGCGAGAACTGGCCAGACGATTCCGATCCGAGGGTGACCCTGCCGCTTCTTCCTCTTCTGCCCTGCTGAACCGATCTAT*<br>*CGCCGCCAAGCTCCTGGCTCGACTGCACAACGGCGGCTACGTGCTCCCCGCCGACATTTTTGCTAACCTGCCTAACCCTCCTGATCCATTCTTTACT*<br>*CGAGCCCAGATTGACCGAGAGGCCCGAAAGGTGCGAGATGGCATCATGGGCATGCTGTACCTGCAGCGAATGCCCACCGAGTTCGACGTCGCCAT*<br>*GGCCACCGTCTACTACCTGGCCGACCGAAACGTGTCCGGTGAGACTTTTCACCCTTCTGGCGGCCTGCGATACGAGCGAACTCCCACCGGCGGTG*<br>*AGCTGTTCGGACTGCCTTCGCCTGAGCGACTGGCAGAGCTGGTCGGCTCTACCGTGTACCTGATCGGCGAGCACCTCACCGAGCACCTGAACCTG*<br>*CTTGCCCGAGCCTACCTTGAGCGATACGGTGCCCGACAGGTGGTGATGATCGTCGAGACTGAGACTGGTGCCGAGACTATGCGACGACTCCTGCA*<br>*CGACCACGTCGAGGCCGGACGACTGATGACCATTGTGGCTGGTGACCAGATTGAGGCCGCCATTGACCAGGCTATCACCCGATACGGTCGACCCG*<br>*GACCAGTGGTGTGCACCCCTTTCCGACCTCTGCCTACCGTGCCTCTGGTGGGCCGAAAGGACTCTGACTGGTCTACCGTGCTGTCTGAGGCCGAG*<br>*TTCGCTGAGCTGTGCGAGCACCAGCTGACCCACCACTTTCGAGTCGCCCGAAAGATTGCCCTGTCCGACGGCGCCTCTCTGGCCCTGGTGACCCC*<br>*TGAGACTACCGCTACCTCTACCACCGAGCAGTTCGCCCTGGCCAACTTCATCAAGACCACTCTGCACGCTTTCACCGCTACCATCGGCGTCGAGTC*<br>*GGAGCGAACAGCCCAGCGAATCCTGATTAACCAGGTGGACCTGACTCGACGAGCTAGAGCCGAGGAACCCAGAGATCCCCACGAGCGACAGCAAG*<br>*AGCTGGAACGATTCATCGAGGCTGTGCTGCTGGTCACCGCTCCACTGCCACCTGAGGCTGACACTCGATACGCCGGACGAATTCACCGAGGACGA*<br>*GCCATCACCGTGTAA*

Codon-optimized $GAPN_{Sm}$ gene sequence	*ATGACCAAGCAGTACAAGAACTACGTCAACGGCGAGTGGAAGCTGTCCGAGAACGAGATCAAGATCTACGAGCCCGCCTCCGGCGCCGAGCTGGG<br>TTCCGTCCCTGCTATGTCCACCGAGGAGGTCGACTACGTCTACGCCTCTGCCAAGAAGGCCCAGCCCGCCTGGCGAGCTCTCTCTTACATCGAGCG<br>AGCCGCCTACCTCCACAAGGTCGCCGATATCCTGATGCGAGACAAGGAGAAGATTGGCGCCATCCTGTCCAAGGAGGTCGCCAAGGGCTACAAGT<br>CCGCCGTCTCCGAGGTCGTGCGAACTGCCGAGATCATCAACTACGCCGCTGAGGAGGGCCTGCGAATGGAGGGAGAGGTCCTGGAGGGCGGCTC<br>CTTTGAGGCCGCTTCTAAGAAGAAGATTGCCGTTGTGCGACGAGAGCCCGTGGGACTGGTCCTGGCTATCTCTCCCTTTAACTACCCCGTCAACCTG<br>GCCGGTTCCAAGATTGCTCCCGCCCTGATTGCCGGCAACGTCATCGCCTTCAAGCCCCCCACCCAGGGCTCCATCTCCGGTCTGCTCCTCGCCGA<br>GGCCTTCGCTGAGGCTGGACTCCCTGCTGGCGTCTTCAACACCATCACCGGCCGAGGCTCCGAGATCGGCGACTACATCGTCGAGCACCAGGCCG<br>TCAACTTCATCAACTTCACCGGCTCCACCGGAATCGGAGAGCGAATCGGAAAGATGGCCGGCATGCGACCCATTATGCTGGAGCTGGGAGGCAAG<br>GACTCCGCCATTGTCCTCGAGGATGCCGACCTCGAGCTGACCGCTAAGAACATTATCGCTGGTGCCTTCGGTTACTCCGGTCAGCGATGCACCGCC<br>GTCAAGCGAGTCCTGGTTATGGAGTCCGTCGCCGATGAGCTGGTGGAGAAGATCCGAGAGAAGGTCCTGGCCCTGACCATTGGCAACCCCGAGGA<br>CGACGCTGACATTACCCCCCTGATCGACACCAAGTCCGCTGACTACGTCGAGGGTCTGATTAACGATGCCAACGACAAGGGCGCCACCGCCCTGA<br>CTGAGATCAAGCGAGAGGGAAACCTGATTTGCCCCATCCTCTTCGACAAGGTCACCACCGACATGCGACTGGCCTGGGAGGAGCCCTTCGGTCCT<br>GTGCTGCCCATCATCCGAGTTACCTCCGTCGAGGAGGCCATCGAGATCTCCAACAAGTCCGAGTACGGCCTGCAGGCCTCCATCTTCACCAACGAT<br>TTTCCCCGAGCCTTCGGTATCGCTGAGCAGCTGGAGGTCGGCACCGTCCATATTAACAACAAGACCCAGCGAGGCACCGACAACTTCCCCTTCCTC<br>GGCGCCAAGAAGTCCGGCGCTGGCATTCAGGGTGTCAAGTACTCCATTGAGGCCATGACCACTGTCAAGTCCGTCGTCTTCGACATCAAGTAA*
Codon-optimized $GAPN_{Bc}$ gene sequence	*ATGACCACCTCCAACACCTACAAGTTCTACCTGAACGGCGAGTGGCGAGAGTCCTCCTCCGGCGAGACCATCGAGATCCCCTCCCCCTACCTCCAC<br>GAGGTCATCGGCCAGGTCCAGGCCATCACCCGAGGCGAGGTCGACGAGGCTATTGCCTCTGCCAAGGAGGCCCAGAAGTCCTGGGCCGAGGCCT<br>CTCTGCAGGACCGAGCTAAGTACCTGTACAAGTGGGCCGATGAGCTGGTCAACATGCAGGACGAGATCGCCGACATCATCATGAAGGAGGTGGGT<br>AAGGGCTACAAGGATGCCAAGAAGGAGGTCGTGCGAACTGCCGATTTCATCCGATACACCATTGAGGAGGCTCTGCACATGCACGGAGAGTCTATG<br>ATGGGCGACTCGTTCCCTGGTGGAACTAAGTCCAAGCTGGCCATCATCCAGCGAGCCCCCCTGGGTGTCGTCCTCGCTATCGCTCCCTTTAACTAC<br>CCCGTCAACCTGTCCGCCGCCAAGCTGGCTCCCGCTCTGATTATGGGCAACGCCGTCATCTTCAAGCCCGCCACCCAGGGCGCTATCTCCGGTATC<br>AAGATGGTCGAGGCCCTGCACAAGGCCGGCCTCCCTAAGGGTCTGGTCAACGTCGCCACCGGCCGAGGTTCCGTTATTGGCGATTACCTCGTCGA<br>GCACGAGGGTATCAACATGGTCTCCTTCACCGGCGGTACCAACACCGGTAAGCACCTCGCCAAGAAGGCCTCCATGATTCCCCTGGTCCTCGAGCT<br>GGGCGGCAAGGACCCTGGTATTGTCCGAGAGGACGCCGACCTGCAGGACGCTGCTAACCACATCGTCTCTGGAGCCTTCTCCTACTCCGGTCAGC<br>GATGCACCGCCATTAAGCGAGTCCTGGTTCACGAGAACGTGGCCGATGAGCTCGTGTCCCTGCTCAAGGCCCAGGTCGCCGAGCTGTCTGTCGGC<br>TCTCCCGAGCAGGACTCCACCATCGTCCCCCTGATCGACGACAAGTCCGCCGACTTTGTCCAGGGCCTGGTCGACGATGCCGTCGAGAAGGGTGC<br>CACCATTGTTATCGGCAACAAGCGAGAGCGAAACCTGATTTACCCCACCCTGATTGACCACGTGACCGAGGAGATGAAGGTGGCCTGGGAGGAGC<br>CCTTCGGTCCCATTCTGCCTATCATCCGAGTTTCCTCCGACGAGCAGGCCATCGAGATCGCCAACAAGTCGGAGTTCGGACTCCAGGCCTCCGTGT<br>TCACCAAGGATATCAACAAGGCCTTCGCCATCGCCAACAAGATCGAGACCGGATCTGTCCAGATCAACGGTCGAACCGAGCGAGGACCCGACCACT<br>TCCCCTTCATCGGTGTCAAGGGCTCCGGCATGGGCGCCCAGGGTATCCGAAAGTCCCTCGAGTCCATGACCCGAGAGAAGGTCACCGTCCTGAAC<br>CTGGTGTAA*
ACC1<br>(KEGG: YALI0C11407p)	*ATGCGACTGCAATTGAGGACACTAACACGTCGGTTTTTCAGTATGGCTTCAGGATCTTCAACGCCAGATGTGGCTCCCTTGGTGGACCCCAACATTC<br>ACAAAGGTCTCGCCTCTCATTTCTTTGGACTCAATTCTGTCCACACAGCCAAGCCCTCAAAAGTCAAGGAGTTTGTGGCTTCTCACGGAGGTCATACA<br>GTTATCAACAAGGTCCTCATCGCTAACAACGGTATTGCCGCAGTAAAGGAGATCCGTTCAGTACGAAAATGGGCCTACGAGACCTTTGGCGACGAGC<br>GAGCAATCTCGTTCACCGTCATGGCCACCCCCGAAGATCTCGCTGCCAACGCCGACTACATTAGAATGGCCGATCAGTACGTCGAGGTGCCCGGAG<br>GAACCAACAACAACAACTACGCCAACGTCGAGCTGATTGTCGACGTGGCTGAGCGATTCGGCGTCGATGCCGTGTGGGCCGGATGGGGCCATGCC<br>AGTGAAAATCCCCTGCTCCCCGAGTCGCTAGCGGCCTCTCCCCGCAAGATTGTCTTCATCGGCCCTCCCGGAGCTGCCATGAGATCTCTGGGAGAC<br>AAAATTTCTTCTACCATTGTGGCCCAGCACGCAAAGGTCCCGTGTATCCCGTGGTCTGGAACCGGAGTGGACGAGGTTGTGGTTGACAAGAGCACC<br>AACCTCGTGTCCGTGTCCGAGGAGGTGTACACCAAGGGCTGCACCACCGGTCCCAAGCAGGGTCTGGAGAAGGCTAAGCAGATTGGATTCCCCGT<br>GATGATCAAGGCTTCCGAGGGAGGAGGAGGAAAGGGTATTCGAAAGGTTGAGCGAGAGGAGGACTTCGAGGCTGCTTACCACCAGGTCGAGGGA<br>GAGATCCCCGGCTCGCCCATCTTCATTATGCAGCTTGCAGGCAATGCCCGGCATTTGGAGGTGCAGCTTCTGGCTGATCAGTACGGCAACAATATTT<br>CACTGTTTGGTCGAGATTGTTCGGTTCAGCGACGGCATCAAAAGATTATTGAGGAGGCTCCTGTGACTGTGGCTGGCCAGCAGACCTTCACTGCCAT<br>GGAGAAGGCTGCCGTGCGACTCGGTAAGCTTGTCGGATATGTCTCTGCAGGTACCGTTGAATATCTGTATTCCCATGAGGACGACAAGTTCTACTTC<br>TTGGAGCTGAATCCTCGTCTTCAGGTCGAACATCCTACCACCGAGATGGTCACCGGTGTCAACCTGCCCGCTGCCCAGCTTCAGATCGCCATGGGT<br>ATCCCCCTCGATCGAATCAAGGACATTCGTCTCTTTTACGGTGTTAACCCTCACACCACCACTCCAATTGATTTCGACTTCTCGGGCGAGGATGCTGA<br>TAAGACACAGCGACGTCCCGTCCCCCGAGGTCACACCACTGCTTGCCGAATCACATCCGAGGACCCTGGAGAGGGTTTCAAGCCCTCCGGAGGTA<br>CTATGCACGAGCTCAACTTCCGATCCTCGTCCAACGTGTGGGGTTACTTCTCCGTTGGTAACCAGGGAGGTATCCATTCGTTCTCGGATTCGCAGTT<br>TGGTCACATCTTCGCCTTCGGTGAGAACCGAAGTGCGTCTCGAAAGCACATGGTTGTTGCTTTGAAGGAACTATCTATTCGAGGTGACTTCCGAACC<br>ACCGTCGAGTACCTCATCAAGCTGCTGGAGACACCGGACTTCGAGGACAACACCATCACCACCGGCTGGCTGGATGAGCTTATCTCCAACAAGCTG<br>ACTGCCGAGCGACCCGACTCGTTCCTCGCTGTTGTTTGTGGTGCTGCTACCAAGGCCCATCGAGCTTCCGAGGACTCTATTGCCACCTACATGGCTT<br>CGCTAGAGAAGGGCCAGGTCCCTGCTCGAGACATTCTCAAGACCCTTTTCCCCGTTGACTTCATCTACGAGGGCCAGCGGTACAAGTTCACCGCCA<br>CCCGGTCGTCTGAGGACTCTTACACGCTGTTCATCAACGGTTCTCGATGCGACATTGGAGTTAGACCTCTTTCTGACGGTGGTATTCTGTGTCTTGTA<br>GGTGGGAGATCCCACAATGTCTACTGGAAGGAGGAGGTTGGAGCCACGCGACTGTCTGTTGACTCCAAGACCTGCCTTCTCGAGGTGGAGAACGA<br>CCCCACTCAGCTTCGATCTCCCTCTCCCGGTAAGCTGGTTAAGTTCCTGGTCGAGAACGGCGACCACGTGCGAGCCAACCAGCCCTATGCCGAGAT<br>TGAGGTCATGAAGATGTACATGACTCTCACTGCTCAGGAGGACGGTATTGTCCAGCTGATGAAGCAGCCCGGTTCCACCATCGAGGCTGGCGACAT<br>CCTCGGTATCTTGGCCCTTGATGATCCTTCCAAGGTCAAGCATGCCAAGCCCTTTGAGGGCCAGCTTCCCGAGCTTGGACCCCCCACTCTCAGCGG<br>TAACAAGCCTCATCAGCGATACGAGCACTGCCAGAACGTGCTCCATAACATTCTGCTTGGTTTCGATAACCAGGTGGTGATGAAGTCCACTCTTCAG<br>GAGATGGTTGGTCTGCTCCGAAACCCTGAGCTTCCTTATCTCCAGTGGGCTCATCAGGTGTCTTCTCTGCACACCCGAATGAGCGCCAAGCTGGAT<br>GCTACTCTTGCTGGTCTCATTGACAAGGCCAAGCAGCGAGGTGGCGAGTTTCCTGCCAAGCAGCTTCTGCGAGCCCTTGAGAAGGAGGCGAGCTCT<br>GGCGAGGTCGATGCGCTCTTCCAGCAAACTCTTGCTCCTCTGTTTGACCTTGCTCGAGAGTACCAGGACGGTCTTGCTATCCACGAGCTTCAGGTTG<br>CTGCAGGCCTTCTGCAGGCCTACTACGACTCTGAGGCCCGGTTCTGCGGACCCAACGTACGTGACGAGGATGTCATTCTCAAGCTTCGAGAGGAGA<br>ACCGAGATTCTCTTCGAAAGGTTGTGATGGCCCAGCTGTCTCATTCTCGAGTCGGAGCCAAGAACAACCTTGTGCTGGCCCTTCTCGATGAATACAA<br>GGTGGCCGACCAGGCTGGCACCGACTCTCCTGCCTCCAACGTGCACGTTGCAAAGTACTTGCGACCTGTGCTGCGAAAGATTGTGGAGCTGGAAT<br>CTCGAGCTTCTGCCAAGGTATCTCTGAAAGCCCGAGAGATTCTCATCCAGTGCGCTCTGCCCTCTCTAAAGGAGCGAACTGACCAGCTTGAGCACAT<br>TCTGCGATCTTCTGTCGTCGAGTCTCGATACGGAGAGGTTGGTCTGGAGCACCGAACTCCCCGAGCCGATATTCTCAAGGAGGTTGTCGACTCCAA<br>GTACATTGTCTTTGATGTGCTTGCCCAGTTCTTTGCCCACGATGATCCCTGGATCGTCCTTGCTGCCCTGGAGCTGTACATCCGACGAGCTTGCAAG<br>GCCTACTCCATCCTGGACATCAACTACCACCAGGACTCGGACCTGCCTCCCGTCATCTCGTGGCGATTTAGACTGCCTACCATGTCGTCTGCTTTGT*

	ACAACTCAGTAGTGTCTTCTGGCTCCAAAACCCCCACTTCCCCCTCGGTGTCTCGAGCTGATTCCGTCTCCGACTTTTCGTACACCGTTGAGCGAGA<br>CTCTGCTCCCGCTCGAACCGGAGCGATTGTTGCCGTGCCTCATCTGGATGATCTGGAGGATGCTCTGACTCGTGTTCTGGAGAACCTGCCCAAACG<br>GGGCGCTGGTCTTGCCATCTCTGTTGGTGCTAGCAACAAGAGTGCCGCTGCTTCTGCTCGTGACGCTGCTGCTGCTGCCGCTTCATCCGTTGACAC<br>TGGCCTGTCCAACATTTGCAACGTTATGATTGGTCGGGTTGATGAGTCTGATGACGACGACACTCTGATTGCCCGAATCTCCCAGGTCATTGAGGAC<br>TTTAAGGAGGACTTTGAGGCCTGTTCTCTGCGACGAATCACCTTCTCCTTCGGCAACTCCCGAGGTACTTATCCCAAGTATTTCACGTTCCGAGGCC<br>CCGCATACGAGGAGGACCCCACTATCCGACACATTGAGCCTGCTCTGGCCTTCCAGCTGGAGCTCGCCCGTCTGTCCAACTTCGACATCAAGCCTG<br>TCCACACCGACAACCGAAACATCCACGTGTACGAGGCTACTGGCAAGAACGCTGCTTCCGACAAGCGGTTCTTCACCCGAGGTATCGTACGACCTG<br>GTCGTCTTCGAGAGAACATCCCCACCTCGGAGTATCTCATTTCCGAGGCTGACCGGCTCATGAGCGATATTTTGGACGCTCTAGAGGTGATTGGAAC<br>CACCAACTCGGATCTCAACCACATTTTCATCAACTTCTCAGCCGTCTTTGCTCTGAAGCCCGAGGAGGTTGAAGCTGCCTTTGGCGGTTTCCTGGAG<br>CGATTTGGCCGACGTCTGTGGCGACTTCGAGTCACCGGTGCCGAGATCCGAATGATGGTATCCGACCCCGAAACTGGCTCTGCTTTCCCTCTGCGA<br>GCAATGATCAACAACGTCTCTGGTTACGTTGTGCAGTCTGAGCTGTACGCTGAGGCCAAGAACGACAAGGGCCAGTGGATTTTCAAGTCTCTGGGC<br>AAGCCCGGCTCCATGCACATGCGGTCTATCAACACTCCCTACCCCACCAAGGAGTGGCTGCAGCCCAAGCGGTACAAGGCCCATCTGATGGGTAC<br>CACCTACTGCTATGACTTCCCCGAGCTGTTCCGACAGTCCATTGAGTCGGACTGGAAGAAGTATGACGGCAAGGCTCCCGACGATCTCATGACTTG<br>CAACGAGCTGATTCTCGATGAGGACTCTGGCGAGCTGCAGGAGGTGAACCGAGAGCCCGGCGCCAACAACGTCGGTATGGTTGCGTGGAAGTTTG<br>AGGCCAAGACCCCCGAGTACCCTCGAGGCCGATCTTTCATCGTGGTGGCCAACGATATCACCTTCCAGATTGGTTCGTTTGGCCCTGCTGAGGACC<br>AGTTCTTCTTCAAGGTGACGGAGCTGGCTCGAAAGCTCGGTATTCCTCGAATCTATCTGTCTGCCAACTCTGGTGCTCGAATCGGCATTGCTGACGA<br>GCTCGTTGGCAAGTACAAGGTTGCGTGGAACGACGAGACTGACCCCTCCAAGGGCTTCAAGTACCTTTACTTCACCCCTGAGTCTCTTGCCACCCTC<br>AAGCCCGACACTGTTGTCACCACTGAGATTGAGGAGGAGGGTCCCAACGGCGTGGAGAAGCGTCATGTGATCGACTACATTGTCGGAGAGAAGGA<br>CGGTCTCGGAGTCGAGTGTCTGCGGGGCTCTGGTCTCATTGCAGGCGCCACTTCTCGAGCCTACAAGGATATCTTCACTCTCACTCTTGTCACCTGT<br>CGATCCGTTGGTATCGGTGCTTACCTTGTTCGTCTTGGTCAACGAGCCATCCAGATTGAGGGCCAGCCCATCATTCTCACTGGTGCCCCCGCCATCA<br>ACAAGCTGCTTGGTCGAGAGGTCTACTCTTCCAACTTGCAGCTTGGTGGTACTCAGATCATGTACAACAACGGTGTGTCTCATCTGACTGCCCGAGA<br>TGATCTCAACGGTGTCCACAAGATCATGCAGTGGCTGTCATACATCCCTGCTTCTCGAGGTCTTCCAGTGCCTGTTCTCCCTCACAAGACCGATGTG<br>TGGGATCGAGACGTGACGTTCCAGCCTGTCCGAGGCGAGCAGTACGATGTTAGATGGCTTATTTCTGGCCGAACTCTCGAGGATGGTGCTTTCGAG<br>TCTGGTCTCTTTGACAAGGACTCTTTCCAGGAGACTCTGTCTGGCTGGGCCAAGGGTGTTGTTGTTGGTCGAGCTCGTCTTGGCGGCATTCCCTTCG<br>GTGTCATTGGTGTCGAGACTGCGACCGTCGACAATACTACCCCTGCCGATCCCGCCAACCCGGACTCTATTGAGATGAGCACCTCTGAAGCCGGCC<br>AGGTTTGGTACCCCAACTCGGCCTTCAAGACCTCTCAGGCCATCAACGACTTCAACCATGGTGAGGCGCTTCCTCTCATGATTCTTGCTAACTGGCG<br>AGGCTTTTCTGGTGGTCAGCGAGACATGTACAATGAGGTTCTCAAGTACGGATCTTTCATTGTTGATGCTCTGGTTGACTACAAGCAGCCCATCATGG<br>TGTACATCCCTCCCACCGGTGAGCTGCGAGGTGGTTCTTGGGTTGTGGTTGACCCCACCATCAACTCGGACATGATGGAGATGTACGCTGACGTCG<br>AGTCTCGAGGTGGTGTGCTGGAGCCCGAGGGAATGGTCGGTATCAAGTACCGACGAGACAAGCTACTGGACACCATGGCTCGTCTGGATCCCGAG<br>TACTCCTCTCTCAAGAAGCAGCTTGAGGAGTCTCCCGATTCTGAGGAGCTCAAGGTCAAGCTCAGCGTGCGAGAGAAGTCTCTCATGCCCATCTAC<br>CAGCAGATCTCCGTGCAGTTTGCCGACTTGCATGACCGAGCTGGCCGAATGGAGGCCAAGGGTGTCATTCGTGAGGCTCTTGTGTGGAAGGATGCT<br>CGTCGATTCTTCTTCTGGCGAATCCGACGACGATTAGTCGAGGAGTACCTCATTACCAAGATCAATAGCATTCTGCCCTCTTGCACTCGGCTTGAGTG<br>TCTGGCTCGAATCAAGTCGTGGAAGCCTGCCACTCTTGATCAGGGCTCTGACCGGGGTGTTGCCGAGTGGTTTGACGAGAACTCTGATGCCGTCTC<br>TGCTCGACTCAGCGAGCTCAAGAAGGACGCTTCTGCCCAGTCGTTTGCTTCTCAACTGAGAAAGGACCGACAGGGTACTCTCCAGGGCATGAAGCA<br>GGCTCTCGCTTCTCTTTCTGAGGCTGAGCGGGCTGAGCTGCTCAAGGGGTTGTGA
Codon-optimized mutant *ACS*$^{L641P}_{Se}$ gene sequence	ATGTCCCAGACCCACAAGCACGCCATCCCCGCCAACATCGCCGACCGATGCCTCATCAACCCCGAGCAGTACGAGACCAAGTACAAGCAGTCCATC<br>AACGACCCCGACACCTTCTGGGGCGAGCAGGGAAAGATCCTCGACTGGATCACCCCCTACCAGAAGGTCAAGAACACCTCCTTCGCCCCCGGCAA<br>CGTCTCCATCAAGTGGTACGAGGACGGAACCCTGAACCTGGCCGCTAACTGCCTGGACCGACACCTGCAGGAGAACGGCGACCGAACCGCCATCA<br>TCTGGGAGGGCGATGACGCTTCCCAGTCCAAGCACATCTCCTACCGAGAGCTCCACCGAGACGTTTGTCGATTCGCCAACACCCTCCTGGACCTGG<br>GCATTAAGAAGGGCGACGTCGTCGCTATTTACATGCCCATGGTCCCCGAGGCCGCCGTCGCTATGCTGGCTTGTGCCCGAATTGGCGCCGTCCACT<br>CCGTCATCTTCGGCGGTTTTTCCCCCGAGGCCGTTGCCGGTCGAATTATCGACTCCTCCTCCCGACTGGTTATCACTGCCGACGAGGGCGTTCGAG<br>CCGGCCGATCCATCCCTCTCAAGAAGAACGTTGACGATGCTCTGAAGAACCCCAACGTCACTTCCGTCGAGCACGTCATCGTCCTCAAGCGAACCG<br>GCTCCGACATCGATTGGCAGGAGGGCCGAGACCTGTGGTGGCGAGACCTGATTGAGAAGGCCTCCCCCGAGCACCAGCCTGAGGCTATGAACGC<br>CGAGGACCCCCTGTTCATCCTGTACACCTCCGGCTCCACCGGCAAGCCCAAGGGAGTCCTGCACACCACCGGCGGTTACCTGGTCTACGCCGCCA<br>CCACCTTCAAGTACGTCTTCGACTACCACCCCTCCGACATCTACTGGTGCACCGCTGACGTCGGCTGGGTCACCGGTCATTCCTACCTCCTGTACG<br>GCCCCCTCGCCTGCGGTGCTACTACCCTGATGTTCGAGGGCGTCCCCAACTGGCCTACCCCCGCTCGAATGTGCCAGGTGGTGGATAAGCATCAG<br>GTGAACATCCTCTACACCGCCCCCACCGCCATCCGAGCTCTGATGGCTGAGGGCGACAAGGCTATCGAGGGCACCGACCGATCCTCCCTGCGAAT<br>CCTGGGATCTGTGGGCGAGCCCATCAACCCCGAGGCCTGGGAGTGGTACTGGAAGAAGATTGGTAAGGAGAAGTGCCCCGTCGTTGACACCTGGT<br>GGCAGACCGAGACCGGAGGCTTCATGATTACTCCCCTGCCCGGCGCCATCGAGCTGAAGGCTGGTTCTGCCACCCGACCCTTCTTCGGAGTGCAG<br>CCCGCCCTGGTCGACAACGAGGGTCACCCTCAGGAGGGTGCTACCGAGGGCAACCTCGTCATCACTGATTCCTGGCCCGGCCAGGCCCGAACCC<br>TGTTTGGTGACCACGAGCGATTCGAGCAGACCTACTTCTCCACCTTTAAGAACATGTACTTCTCCGGCGACGGCGCCCGACGAGACGAGGACGGTT<br>ACTACTGGATCACCGGCCGAGTCGACGATGTGCTCAACGTCTCCGGCCACCGACTGGGCACCGCTGAGATCGAGTCCGCCCTGGTGGCTCACCCC<br>AAGATTGCCGAGGCCGCCGTGGTCGGTATTCCCCATGCTATTAAGGGCCAGGCCATCTACGCCTACGTTACCCTGAACCACGGCGAGGAGCCCTC<br>CCCTGAGCTGTACGCTGAGGTCCGAAACTGGGTGCGAAAGGAGATTGGCCCCCTGGCCACCCCCGACGTTCTGCACTGGACCGATTCCCTGCCCA<br>AGACCCGATCCGGTAAGATCATGCGACGAATCCTGCGAAAGATCGCCGCCGGCGATACCTCGAACCTGGGCGATACCTCCACCCTCGCCGATCCC<br>GGTGTCGTCGAGAAGCCCCTCGAGGAGAAGCAGGCCATCGCCATGCCCTCCTAA
ALD6<br>(KEGG: YALI0A17875p)	ATGTCCTGGGAAACAATCACTCCTCCTACGCCAATCGATACGTTTGACAGCAACTTGCAACGTCTTCGAGACTCTTTCGAGACCGGCAAGCTCGACTC<br>TGTCGACTACCGTCTCGAGCAGCTGCGAACCCTGTGGTTCAAGTTCTACGACAACCTCGACAACATCTACGAGGCGGTCACCAAGGATCTCCATCGA<br>CCCAGGTTCGAAACCGAGCTCACCGAGGTACTGTTTGTTCGAGACGAGTTCTCCACCGTCATCAAGAACCTGCGAAAGTGGGTCAAGGAAGAAAAG<br>GTGGAGAACCCCGGAGGCCCCTTCCAGTTTGCCAACCCCCGAATCCGACCCGTTCCTCTGGGAGTGGTGCTGGTCATCACTCCCTGGAACTACCCC<br>GTCATGCTCAACATCTCACCTGTGATTGCCGCCATTGCTGCCGGCTGTCCCATCGTGCTCAAGATGTCCGAGCTGTCTCCCCACACTTCCGCTGTTC<br>TTGGCCGAATCTTCAAGGAGGCCCTGGACCCCGGTATCATCCAGGTTGTTTACGGAGGTGTCCCCGAGACCACCGCCCTTCTTACCCAGCATTGGG<br>ACAAGATCATGTACACCGGAAACGGAGCCGTTGGTCGAATCATCGCCCAGGCCGCGGTCAAGAACCTGACTCCTCTAGCTCTTGAGCTTGGTGGCA<br>AGTCACCCGTGTTCATCACTTCCAACTGCAAGAGCGTTATGACGGCCGCTCGGCGAATCGTGTGGGGCAAGTTTGTCAACGCCGGCCAGATCTGTG<br>TCGCTCCAGACTACATTCTGGTTGCTCCCGAAAAGGAGGCCGAGCTCGTCGCTTGTATCAAGGAGGTGCTCCAAGAACGATACGGCTCCAAGAGAG<br>ACGCCCACCACCCCGATCTGTCCCATATCATTTCCAAGCCCCATTGGAAGCGTATTCACAACATGATCGCCCAGACCAAGGGAGACATCCAGGTGGG<br>TGGACTCGAGAACGCCGACGAAGACCAAAAGTTCATCCAGCCCACAATCGTCTCCAACGTTCCAGATGACGACATTCTCATGCAGGACGAGATTTTC

	GGACCCATCATCCCCATCATCAAGCCCCGAACCCTCGGCCAGCAGGTTGATTACGTCACAAGAAACCATGACACCCCCCTGGCCATGTACATCTTCT<br>*CTGACGACCCCAAGGAGGTGGACTGGCTACAGACCCGAATCCGAGCTGGTTCTGTAAACATCAACGAGGTCATTGAGCAGGTCGGACTGGCCTCTC*<br>*TGCCTCTCAGTGGAGTTGGAGCTTCCGGAACCGGAGCATACCATGGAAAATTCTCCTTCGATGTCTTCACCCACAAGCAGGCCGTTATGGGACAGCC*<br>*CACCTGGCCCTTCTTTGAATACCTCATGTATTACCGGTACCCTCCTTACTCCGAGTACAAGATGAAGGTGCTCCGAACCCTGTTCCCACCGGTTCTGAT*<br>*TCCTCGAACCGGCCGACCCGACGCTACTGTTCTTCAGCGAGTTCTCGGC*AACAAGCTGCTTTGGATCATTATTGCCGCCCTTGTTGCGTACGCCA<br>AACGAAATGAGCTGCTCATCACCATTGCTCAGATTATGTCGGTGTTTATTAAGTAG
MLS1<br>(KEGG:<br>YALI0D19140p)	*ATGACAGTCAACTCCACTTTTAGATCGGCATCAACTTCCCCGAAACTGGGCAAAACCAGCCAGGCAGACATCCTGAGCCCCGAGGCCCAAAAGTTCC*<br>*TGGTTGAACTCCACAGCAACTTCAACCAGCGACGTCTGGAGCTCCTTGATCTGCGTCAGAAGAACCAGCTCAAGCTCGATGCAGGCGAAATCCCCA*<br>*CGTATCCCACGGAAACAGCAGACATCCGAGCAGACAAGTCGTGGACAGGTCCATCTCTGGCTCCCGGTCTCCATGACCGACGGGTCGAAATCACTG*<br>*GCCCCCCAGACCGAAAGATGATCATCAACGCCCTCAACACAAACGTCGCCACCTACATGTCCGATTTCGAGGACTCCCAAGCCCCCACCTGGGACA*<br>*ACTGTCTCGATGGGCAAGTCAACCTGTACGATGCCATCCGAAACCAGGTTGATTTCGACACAGAGAAGAAACCCTACAAGCTGACTACAAAGAAGTG*<br>*GACCGAGGGGACCTACTCTAGAGGCTCCACGGACACTCGACCCACTCTTTTGGTGCGCCCTAGAGGCTGGCACATGCTCGAAAGCCATGTTCAGAT*<br>*CGATGGACAGAGCATGTCTGGGTCTCTGTTCGACTTTGGACTCTTCTTCTTCAACAACGCCAAGGCTCTGATTGAGGCTGGCCGAGGCCCTTACTTTT*<br>*ACCTCCCCAAGATGGAGCATTATCTCGAGGCTCGACTCTGGAATGATGTCTTTGTTTTCTCTCAAAACTACTGCGGAATCCCCCAGGGCACCATTCGA*<br>*GCTACTTGTCTGATTGAGACTCTTCCTGCAGCTCTGCACATGGAGGAGATCATCTACGAGCTGCGAGATCACTCTGCCGGCCTCAACTGTGGTCGAT*<br>*GGGACTACATGTTCTCAGTTATCAAGCGGTTCCGAAACCAGCCCGAGAAGCTGCTTCCTGACCGAAAGATGATCACCATGACCGTTCCCTTCATGAAC*<br>*GCTTACGTGACTCGTCTGGTTCACGTGTGTCACAAACGAAAGGTGCATGCCATGGGAGGTATGGCTGCCATCATTCCTCTCAAGGATGCTGCGGAGA*<br>*ACGCCCTTGCCATGGAGAAGGTCAAGGCTGACAAACACAGAGAGGCCTCTGCAGGCTGTGACGGTACCTGGATCGCTCATCCCGGTTTGGCTGAGA*<br>*CTGCCACCAAGGAGTTTGACGAGTTGATGCCAGGGGAAAACCAATTTGATTTCGTCGGAGAGGACGTTCCCTCCGAGAAGCTGTTGGATACTACCAT*<br>*TGAAGGCTTTGCCATCACCAAGGAGGGTCTTCAGGAGAATGTCTACATTGGTCTGCGCTACATGGAGGCATGGCTGCGAGGGTTGGGATGTGTGCC*<br>*CATCAACAACCTCATGGAGGATGCTGCTACTGCCGAGGTTTCTCGTGCCCAGCTGTGGCAGTGGACCAAGCACGGCAAGTTCACCAAGGAGGAGGT*<br>*ATTGGAGATGATTTCCCAGGAGGCCGAGAAGCTGGGAAACACCGACTCTGTCAAGCGAGCAGGCGAGTTGCTGGGATCTGAGATTGGCGGCGATTT*<br>*TGCAGAGTTCCTCACCGATCTGCTGTATCCTGATCTGGTTGAACAGTAG*
MLS1 up homology arm 1000bp	*AGGAGGCGCAGGGAGCATCTGAAAGATTAATAAGCACACGTTCGATCTAGCACATGTTTGATCTAGCACGTTCGATCTAGCACGTTCGATCTAGCAC*<br>*GTTCGATCTAGCACCACACTTGATCAATAAACGCCCATAACCACTTGCTGCCTGTACTTGGCCCCCACACATATCTCCGCTTCACTGAGTCAATTCAC*<br>*TGAGTCAAGCACTCCCAATTCTGGGTGTATCTATATGCAGGGTCTCACCGTACGTTAGACGCAGTTTTTGTATGATGTTCCGTTGGTCCGGAGAGAG*<br>*AGAGACCTAACAGTGCCTCATCTAAGAGATACTGCAATTCTCAAGGAGGGTTGGTGTAGCACAGTACATACTGTATCATGGTCGCAGTTGTACTACC*<br>*GTACCGTAATTCACTAGAGCTACAAGCCCCACCCACCAGATAACACCTGGGTATTCTTGGCCATATTGGTTGCAAGTGCACTGTTTCGCTTGAGATCC*<br>*ACCCGACCCTCTCCGAAAACCGGATCATATATCCGTCGCCTGTCTCCCACCTGTATCTTTGCGCCGCCGCCAGCAATTTTAGACGCGGGAGTCTGG*<br>*AGATGAGCGATAAGTTAATCGGCCGATATTCGGATGGATGGAAGATGGCTTGTGTGGCGTTTGAGACAACGTAAAAGGGGTGGATTTTTGGCTGACA*<br>*GGGTAAAGCGACCACGACAAGCCCGAATCTGGTGTCAGAATCGCCCTCACGGGTCCAGATCCCGAGTCGGACACCCCCACTTGTCCCCCACTTTTT*<br>*CTTTTAAACTCCCGTCACTCCATGCCGCCAAAATAAAAAAAACTAAACTCAAGGTTGCTGATCTCATGCACCCCGGATAACAGGTCCGTTTCTCCGTT*<br>*CATGTACCATGACACACAGATACCAAAGATGTATATATAGGTAGCGGGCCGTCTACGTTCAGCCCACAAACCTCAAACGTCGCTCCATTTACACACAC*<br>*ACACACACACACACACACACACACAAAC*
MLS1 down homology arm 1000bp	*GTGGGATGACGAAGAGTATTATTTAAGTAACGCGTTATGATTTAGAGAGGACTGCCGGTTGGTGGAGAAAAGGGGATGTAAAATTTTAACAAACAGC*<br>*AGGTACAGTACATACTGTACTTGTACTTGTACTATAGATGTATTCGCTAATGTGAGATTAATCCTGTTTATCTACCTGCACGGTCCGGGCTTACACATC*<br>*TTCCTTTTTCTTGAAAGCCCCGTTGACATAGAGTGCAATTCCCGCTAGGAACACGACCATGCCTGTAATAGTGCCGCGGTTAGTAGCGACTCCATAG*<br>*ATGGTAATAGGCGTTATGGAGTTGATGTCCGTCCAGGAGTAAGTGGATATTTCGTCTTCGTCCATTCCGATGTTGGTGTTGACGATAACGATGATGAA*<br>*GAGTACATCAAGAGATATCAAGAAGGTTCCCTCATCGACACACAGATACCTTTTGTTTTCGGAATCCTTCTATCAGAGCCGTACGGATCTACAAGGTA*<br>*AATACAGTATCTACTCGTGAAACGCTTCCATGGACTGCTTCTTTGTGAAAGGGCGGTGCTAGGGACAAGCAGAATCTCAGATTTACAAAGATTATACC*<br>*CAGTGTCTGTATTTCACCCCCTTTTTTTTGTACGAGCTTGTGCTTCCAGATATATCGCGATATTGTCCATTCTGACGTTCGGCTGTTCTTATCTCGCAT*<br>*GGGGATGCACTATCCAAGCACCTGGTTGTGTCGCCTGTCGTAAATTGTGTTTTGATCTGGACCGTATGTAAACCTCCAAGTGAGCATTTCAAGACGC*<br>*GAACACTTGTCTAACGTGACGGGGAACAGCAGATATATCAGATAACTACATTTTTTCCAGTCTGTCACATCTCTCTTGGATACGGTAGACATTACAGTG*<br>*CTTCACCGATATGGAAGTAGAACTCATCAGAACAGAACTGTTACTTCTCTGACATCCGTTTGATAGAGTCGTACAGAATCATTCTGCTACAAGTATTGT*<br>*AACCTCCTACTATCGAAGA*
CIT2<br>(KEGG:<br>YALI0E00638p)	*ATGATCCCTCTTCGAACCGCCCGTGTTGCCCGAACCTCCGTGTCCTCCATGGTCCAGAAGCGATTTGCTTCTGACCTCAAGGGCGCCCTCAAGGAG*<br>*GCCATCCCCGCCAAGCTGGAGCTCTTCAAGAAGGTCAAGTCCGAGTACTCCCAGAAGTCTCTCGGTGATTGCAAGGTCGAGAACCTGCTCGGAGGC*<br>*ATGCGAGGCCTCAAGTGCATGCTCTGGGAGGGCTCCGTTCTTGACGCTGATGAGGGTATCCGATTCCACGGCAAGACCATCAAGGAGTGCCAGGA*<br>*GGTGCTCCCCAAGGCCGTTGAGGGCGGCGAGATGCTCCCCGAGTCCATGCTGTGGTTCCTCTTCACCGGCAAGGTTCCCACTGAGGAGCAGGTCC*<br>*GAGGCCTGTCTCGAGAGCTCGCTGAGAAGGGCGAGGTCCCCGAGTTTGTCAACAAGATGCTCGACAACCTGCCCCCTACCCTGCACCCCATGACC*<br>*CAGTTCTCCATGGCCGTGTCTGCCCTTAACCACGACTCCAAGTTCGCCAAGGCCTACGAGCGAGGTATCCCCAAGTCCGAGTACTGGGAGTACACC*<br>*TTCGATGACTCCATCGACCTCATTGCCAAGCTGCCCCAGATTGCCGCCAAGATCTACCAGAACTCTTACCTTGGTGGAGGACCTCTCCCCGGCAAGA*<br>*TTGATCTCAACCGAGACTGGTCTTACAACTACGCCGCTATGATGGGCAAGGGCGATGAGACCGGCTTCGTTGATCTCCTGCGACTCTACTTTGCTCT*<br>*CCACGGTGACCACGAGGGAGGAAACGTCTCTGCTCACGCCACCCACCTGGTTGGATCCGCTCTGTCCGATCCTTACCTGTCCTACTCTGCTGGTCT*<br>*CCAGGGTCTTGCTGGTCCTCTCCACGGCCTCGCCGCCCAGGAGGTCCTGCGATTCATCCTCGACATGGAGAAGAACGTCCCCAAGGGCTACTCCA*<br>*ACGAGGACCTCGTCAAGTACCTGTGGTCTGTCCTCAAGTCTGGCCGAGTCATCCCCGGTTACGGCCACGCCGTTCTGCGAAAGCCTGACCCTCGAT*<br>*TCGAGGCTCTGATGAACTTTGCCAACTCTCGACAGGAGATCCGAGACGACCCCGTCTTCCAGCTCGTTTCCCGAACCTCCGAGGTTGCCACTGGCG*<br>*TCCTCACCGAGCACGGCAAGACCAAGAACCCCCACCCCAACGTCGACTCCTCTTCCGGTGTCCTCTTCCACCACTACGGTATCCACGAGACTCTGT*<br>*ACTACACCGTCACCTTCGGTGTGTCCCGAGCTCTTGGTCCTCTGCCTCAGCTGGTCTGGGACCGAATTCTGGGTCTTCCCATTGAGCGACCCAAGT*<br>*CTCTCTCTCTCGAGGAGATTATTAAGGTCGCCAAATAA*

CIT2 up homology arm 1000bp	*GGTATTATTGTGTTTTGCATCACCAATTGCTGCCATACTGGGTCGCAATAAATATCTCCAAGCCGGAAATATAGGGGTGTCCGGTATATCCGGAGCCGCACATCTCTTCTGATTCCACTCGGTGTTGTGTATGGTCTGTTCCAGCTCTGCCCTGGTTACCCTACCGGCCCAACAAGGTGGTCGAGGCATCAAACCTGCACCCCACAAAAACGTGGGGAAATGGGCCGAGATGACCGATCTAGACTATAGTGTGAATTATATATCAATTGGGTAGGGAAAAGGAGAGAATCGTTTCCTCGAGGCTGCACAGCCGCAGAAACGTATCTGGTTTAATCCCATTGCTTCTCACACACATTCTACAAACAGTCGTCGATCCTAAAACCCTACCCCCGCGTACACCTCAATTGTATTTCACGTTTTCCTCCTCGAGGTCTAACACTCTCAAACAATCTTAGTGTACTCCATGTGATGGGCTCCGTGAGTACAGGAGACAATCATGGGGAAACGCGCCGAATTTCTGTCACCGGTCAAATAAAAGATCCATTGCCAGGAATTTGCAGGCAATGAATAATAATGTCAGGCGCGGGGCCCTTTGTTGCAAAGCCCTCCGAGAAAGCCGGGTCGGACGTGTCCAAACTCAAACGTCAGAGTTTATGGAGAAACGGATATTGGAGGAGTTGGAGCCCACTGGTTATACTGTAATTGGCTGATCTGAGAGCCCTAAAAAAGACACATAAGCAAAATTCCCCATCAATTGCTCTGTTTCCAGCTTTACTTATCTTTCCATCTGTCCCACACACCATCCTATACTCATCCGTCGTCTCGGCTCACCCCCGGCATTCACATTCACGCATACAAAGACAAGGTTAACCGGAGGCCCGTTTTGTGCTTTTGGCTATTGGGTCTGATTGCCACGAGCCAGCTTTATATAAGGAAGTGAGCCTGCCCGCTCTGAACAAGGTCTAGAGTCTTCGTAACTAACCCCACACACATCAACACA*
CIT2 down homology arm 1000bp	*GCGAAGTATAGTTGATATGATTTACGATGTTTTGAGCAAGCACAAGCACGAGATGCTTGTAAGATTGTAGATACAGTACCTGCATGAACTAGAATTTGATTCGTGGCAATAGTATCCACCCCGAAAAAGACCCGAAAGACCCGGCACAAATGATCCGGTCTTCTCTGTGTCTTATGCACCAACTCACATGTTTCTCCCCAACTTTGTGAGACTCGGATGTGGCATTGTGGCACAAGTGAGACAGTGAGCCACGGTAAGTTAACGCGCTTCGGTAGTGGCTGTTTTGAGAGAAATTGCACTGTGAACAGTAGCTCTTGTACATACTTGTACAGTACTGTATATAAATTTAGTAGTTCCGTGTGCAGTACACACATGTGCGTTACATTAGCACCGCGGCGGTATGGAGATTTGCTAAGGGTGTATTATCACGCATGAATGATGCTTGCTCCGGATATATGGCATGCACCGGATATATGGCGTGCACCGTATATCGTGAGCTCTCACGCTCACGATATAGCTCACGATATACGGTATTATTGAAAGCCAGTTTTGTGTACTTTCTGGGATATTCCCATAATCATGTTACGTAGTCTTCGTCGGTGTAATTTTCGTACACTGACTAAAGTAAAGTCCAATTCATACTCTATCAAAAGTTATTCATAACAACAGGCTATGCTCACATCGATCTTATTAATTATACAGTAACACACATTTTTTATATTTTTAGAATCACAATACTTTGCTTAAAAAAACAGTAAGATATGGATGCTCAACCAGACCCATTCAGTCTCTCACAGACTCCATCAAGTAACGACTCTCACTCATTCGTCTCAAATGCTTCCCCAAACGATCACTGGAACGGTGAGAAACAAGGGGATGAGCGTGAAGCATTTCGCAGAGATATAAACGTTCTGTATGCCTATTTTATCTCTGGACAAGAATGATTGCAGTAACCAAAGCGTTACGGACCATAATGACCCCAATGAAGGCGTCGGCACCT*
MMSDH (KEGG: YALI0C01859p)	*ATGCTCAGCAGATCGACCCGACTGGCTCGAGTGGTCCAGACCTCCACCCGGCTCTACTCCACTGCCTACCCCACTACCCATGCGAAAATCGAGAACCCCGTGGACACCAAGGCGTTCATCAACGGCCAAGCAGTCGCCTCCAAGGCTACCACCTGGTTCGATCTCCATGACCCCGCAACAAACAACCTGGTGACGCGAGTCCCCCAGTCCACCGACGAGGAGATGCGACAGGCCGTCGAGGCCGCCCAGAAGGCCTTCCTCACCTGGAAAGACACCTCCATCATGCACAGACAACAGGTGGCTTTCAACTTTGTGCGTCTGATCCGAGACAACTGGGACCGACTCGCCGCCTCCATCACCCTCGAGCAGGGCAAGACCTTTGCCGATGCCAAGGGAGACGTGCTCCGTGGCCTTCAGGTGGCCGAGCAGGCCTGCTCCATCCCCAACACCCTCATGGGCGAGTCCCTGGAGGTGGCCAAGAACATGCGAACCGAAATGTACCGTGAGCCTCTCGGTGTCGTCGCCGCCATTGCTCCCTTCAACTTCCCCGCAATGGTGCCCCTCTGGTCCATCCCCCTGGCTCTCGTGACCGGTAACACTCTCATCCTCAAGCCCTCCGAGCGAGATCCCGGCGCTGCTCTCATCCTCGCCGAGCTCATCACCGAGGCCGGTGCCCCTCCCGGAACCGTCAACATTATCCACGGAGGCGCCCCCACCGTCAACTTCATCTGTGACGCCCCCGAGATCAAGGCCATCTCCTTCGTTGGAGGTGATGCTGCCGGCAAGCACATCCATCAGCGAGGCGGAGCCAACGGTAAGCGAGTTCAGGCCAACCTGGGCGCCAAGAACCACTGCGTTCTGATGCCCGATGCTGACAAGAACTTTGCTCTCAACTCCATTGTCGGCGCTGCCTTTGGCGCTGCCGGCCAGCGATGCATGGCTCTGTCCACCCTGGTCACCACCCAGGGCACCGGCTCGTGGATGCCCGAGCTTGTCGAGCGTGCCAAGAAGCTCACCATTAACGGTGGCTTTGAGGCTGACGCCGATCTGGGACCTGTCATCTCCCCCGCCGCCAAGGCCCGATGTGAGGCTCTGATCCAGTCTGCCATCGACGAGGGAGCCACCGTTCTGCTCGATGGCCGAGGACAGCACCCCGCCAAGTACCCCAACGGTAACTTCATTGGCCCCACCATCATCACCGGTGTCAAGCCCGGCATGAAGTGCTACGACCAGGAGATTTTCGGCCCCGTTCTTGTTGTCGTTGATACCACCGATCTTGACGACGCCATTGGTCTCATCAACCGAAACAAGTACGGCAACGGTGCTGCCATCTTCACCCGATCCGGTGCTGCAGGCAACAAGTTCCAGAAGCAGATCCAGGCCGGCCAGATTGGTATCAACGTGCCCATCCCCGTCCCTCTCCCCATGTTCTCCTTCACCGGAAACAAGGGCTCCTTCCTGGGCGACCTCAACTTCTACGGCAAGGCTGGTGTCATGTTCCTGACCCAGTACAAGACCGTGACCACCCAGTGGCGAGACGAGGATGTCACCTCCCAGAAGGCTGATGTTATCATGCCTACCCAGAGCTAG*
MMSDH up homology arm 1000bp	*CCAGACACAGCACGTTTGGTCGGCGGCTAAAAGTGCATTTGCTGTGTTTCTGGAGATACCACGTATCAATACACCATCTCCACGTGCTAAACGATCGGCTAAAGTCCCTATTTCGACCCATACTGGGCCCATTAACCCATTTAACCTGATATATAAACCACAAACCGTCTCAACAAAAGCTGCATCCGCATTTTGGCCGCACCGCACCGGGTCCGATGCGGTGCGGGGTGCATACTACAGAATACGGTGAAATTTCCGTGCCAAAAAAAATTAAAAAAAATTAAAAAAAATCAGTCAAGATTCGCAAGAATACAACTCCCACAGTGAAAGTGACTCCGACGCTAATTACACCTTTTTAACGCCCAATAAATGACCGGCAAAGACAGGTATGTAAGAGCAGCACGTAGTTTGGCGGCTAAAACCACGTCTCCAGCGCCCAAGAGCCCGATTTCGACAAACCCAAAAGCCTATCATTTTGTTGGACAGCTGCCGAGAAATGGCCGGGAGAGACAATTCCAGCACGTCGCAAGCTTTTCGAGGCCACAAAACGGACCTGTGCCTGACGAGCTCGTTTCAATTGAGTGATTCAAGACAGAGTTTGAACCGGTGGGGTGTTTGACGCGAAAAGCTGACTCGTTTGGTATTAGTGCGTACCCCACAGTCCTGTGCGTACCATGTAGACTATATACTATCTACACGGCTATTGGAGTGGTCCGAAGGGGTGTAGTTTAAGGGCGCGGTAGGGGGGATAATGGAAATATGAAAAGTGCGTTTTGATCAGCTCGTCGCCCAGAATCTATTAACGTGTTCATATTTCCCACATTATCCCCCAAAGCCCCCTTTTTGGTGGTCCTTTGCCCCCTCTTTATCCTCTCCATCCTGCATGGTTTAACCTTCGTCTCTCTCACCCCACGTTTACCGCACCCAAGTATATATATACCCCCAGCCTACCCAAAAACTAACATTTCCAACTTTCTCCCTTCACACCTCCGCCCACACA*
MMSDH down homology arm 1000bp	*ACCGTGAGTGAGCAGTCTTGCCGGTGACCCAGTGACGATGTTCTACAGTTCAACATGCCGAGAATGGCTGGACTGTGAAAAGTATATAATGATGCATATGATTGAAATACGAGAAGTGACTGCGCATGCTTGTAGGTACGAATCACGTGATTTTATTGTTGCTAGGGTTTACATCACTCCAACACATTCTCCACAAAAATTGTTAAGTAAATTGGTCACTTTATCAACGGGTGTTCTCGGCTATCGACTGTGCCGTTAACGGGGAAGTATGTTAGCTGCTACCCAAAGGAAAGATTATGTGAGAGTTGCGTGTTTATGTGCATCTCCGGTGCATTTCCGATGCTCTGGCATCGAAAGGAGTCCGTCCAAGTCTCTGAATGACCGGTTTAGTTGAATCCGGTGTTGGGTGTCTCTGCGGTGATCAGTTTCGGCGGTGATTAGTCTGTTGTGATTAGTCTGCTGCGATTAGTCTGCTGTGATTAGTTTGCTGTGATTACAAGAGCTGTTTTGGAGGGGGTAGGTCTTCTGTAGTTCTTCACATCATGTAGGCTACTTGAACTCCGCTGGCAATCTGTGGACTTCTTTGGGGTAGTGGATCTCGAGCAGTTTCCTTCGAACGCTTGATCTGAAGGATGGGTGACAGCTACTGTAAAGGCGCGAACATGCACCCTGGTTGGTATCACTGAGCAGTACTGTAACATCGGACTGGAATGTCGAGGAGGTCGAAAAGTCGTGACATCTTAGAGATGAGTACAGAGCAGTACACTACAGCACCATACACTACAGCACCATACACTACAGCACTGTACAATACAACACTGTACAATACAACACCGTTTTTTATGTCATGTTACTACAGAAGGAATGGTTGGTGGGTGAACTAATTTCATCTAATAACGAGAGTTGAAGTTGTCTCTAGATGACAATAGATGGATTGGTAATACAATAAATATTTGGTATTATGTAATACAGTAAATATTTGACATTATGTAATACAGTA*

HPDH<br>(KEGG:<br>YALI0F02607p)	*ATGCTCTCATCCACTCTCAGAAGCTCCGTCAAGGCCCTGCGACCGCTGCGGGCCTCCCGGGACCTGTCCTACGGCTTCATTGGTCTGGGTCGAATG<br>GGTCTGCCCATGGCCCTCAATTTGCAGAAGAAGCTGCCCGGTGTCGACGCTGACTCCAAGGTCAACGTGTTCGACATGAACAAGGCTGCTCTAGAA<br>CAGATTGCCAAGGACAAGGATCCCAAGGGCGCCCAGCTGGAGGTGCTGGGCTCTGCCGTGGACGTGGCCAGCAAGTCCGATGTCATCTTCACAAT<br>GCTCCCTGAGCCCGTCCATGTCCGAGGAGTCTACACCGACCTGGTCAACAACCTCGGAGACAAGACCTCCAAGATCTTTGTCGACTGCTCCACCAT<br>TGACACTCAGACGTCAATTGAGTGCGGTAAGCTTGTGACCGACAAGGACGCCTCTGCTGCCTTTGTGGACGCCCCCGTCTCCGGAGGTGTCGTGG<br>GAGCCGAGAAGGGCACCCTCACCTTCATGGTCGGGTGCAACAAGGACAAGCAGCAGGTGTACAAGCAGGTGGAGGAGCTGCTCAAGCTCATGGGT<br>GGCCGAATCGTCAACTGCGGCACCCAGGGCCAGGGTCTGGCTGCCAAGCTTGCCAACAACTACCTGTTGGCTGTGACTAACGTTGCCACTGCTGA<br>GGCTTTCCATCTTGCTGAGAAGCTTGGATGCGACCTCAAGCTCTTCTCAGACATTGTCAACTCCTCTTCCGGTCGATCTTGGTCCTCCGAGGTGAAC<br>AACCCCGTCCCAGGTGTCAACCCCGCTACTCCTTCTTCTCGAGACTACGTTGGAGGCTTTGGTATCAACCTGATGCGAAAGGACCTGGGTCTGGCC<br>ATCGATGTGGCCAAGCAGTCTGGATCCTCCATGCTTGTGGCTGACAAGACTTACGAGGTCTATCAAAACATTGAGAAGGGAGACAACTACCAGGGCA<br>AGGACATGTCTGTTATCTACAAGTTCCTCCAGGATCAGGATCAATAA*
HPDH up<br>homology arm<br>1000bp	*TCACGGTGTGAATGAGTAGTTCTTGAACCTGGTGACCTTCAGGTGCACCAATAATAGCTGGAGCGTCATCAATTAAGAGGCTTTGACGCACGTTATAA<br>AAGTACAAAAAGTGGTTAAGTACAAGTACGATATTTTCGACACTGGTGCAAAGTATGCATGTCCACCAAGAAAGCACTACAAATACTGTAGGATCTCC<br>CACTATTACAGTACATACTGTACATGCTGTAGGTATATAATATGCATGCCGATGACTATGTACTGTACTTGTGGAGCGCATGAAGGGAATCAATCTCAC<br>TTACGGGGCTAACATGGTGGGTATATAGATGGATAGGGTGCAACACCCATAATAGTGCTGTACTTGTACACTGGGCAGGACATAATCACACTGTGGG<br>TTCATCACTGACAACCCAGTGCAAGTTATAACTCAGATGTACACACGCACGTATTGTGCACACGCACGTATTGTGACTACGACCGTCAGTAGAAGTAG<br>AAGGTATATCATTCTATGTGTACTCCTAGTTCAATTTGTAACTTCTCGCCAACCCTCTCGGCCCTCCTCTCCAATTAATATACCACAAGCCTGGTCCCT<br>CACCCAACGAAGATGTCCGCTTCGGCGGCAATCGACGAACTCTGTTGAGTGTACATAACGCAAGACTAATGCTGGGTGGATAATGTGGGGGGGATC<br>CCTTACCTGTACGCAGACGCGGCTAAAAATGCCTTTTGGAGCTAGTCCGAGGAGTTGCGAGGATGAATGCTCCTTTGGAGACAGCTTGAATACGATA<br>TTAGGAAATAAAAGTTTCAGCTCCACCTCCAAACACCTGCAGTGGGACCACTATTATGCGGTTATTGATGCATCTCCCCCACACGCGCCCCGAGACT<br>AGCCCGACAAACCCAAAGTTCAAATCTGGGGTGAGCCGCAGGCCAGAGGATAAAGCTAGACCGATCTCTTTTCTGGCACTCCTTTCCAACACACCAA<br>AACCCACCCTAACACATACACAGC*
HPDH down<br>homology arm<br>1000bp	*AACAAATACAAGTTTTAAGCTGACCCAATGAACTGTGAAACGTATTGCATAAACATTTCATGGATCATGACTAAATAATGAATGATGATGTATGACTTAC<br>ACCCACTGGTTAGGCTCACTTGGTAAACATTTATATAACAATGTGGCTGCTGGTTGTAACTGTCTGTGTATTGGAATGTGTGTTGTCTCTATTAGGCAT<br>TTTCTACCAAACGTCTTTCTATGACGGCATGTACCGTTGCAAGTTATTCGATCTACTGTAGATGTTAGATGTTAGTATGTATACGAAGAAGTCCCCGTC<br>AACCTCCACCTCATTGATGTATTTATTATAGTCATACGTTAATGCTTCTATGCTTACAGGTAGAGACCCTCAAGGTTCTCCTCCACCTTGTTCTTCATGA<br>CCCGCTCTTTAGCGGTTCGGAAATCCTCAGCAGTGACCCGCATTCGTCTCTCTCGCAGAGCAAGCAGACCTGCCTCGGTGCACATGGCCTTGATGT<br>CGGCTCCAGAAAGTTCATCCTTGGAAGACACAAACTCCTCGAGATCAACATCGTCGTTGAGGTTCATCTTGGAGGTGTGGATGCCCATAATCTTTCG<br>CTTGGTGGTGGAGTCGGGATTCTCAAACAGAATCTTTCGGTCGATTCGACCAGGTCGAATCAGTGCAGGGTCCAGCGACTCAATCTTGTTTGTAGCC<br>ATAATTACCTTGACGTCTCCTCGGTCGTCGAATCCATCCAGCTGGTTCAGAAGCTCCAGCATGGTCCGCTGCACCTCTCGCTCTCCGCCAGACGTCG<br>ACTCGTATCGTTTGGTTCCAATAGCGTCAATCTCATCGATGAAAACAATGGACGGTGCGTGTTCTGCGGCAATCTGGAAAATCTGTCGACACAGTCG<br>GGGACCGTCTCCCAAGTACTTTTGGATCAGCTCGGATCCAACGATTCGCAAAAATGTAGCAGAAGTCTGGTTGGCGACGGCCTTGGCCAGCAGTGT<br>CTTTCCGGTTCCGGGAGCGCCG*

Table S2. sgRNA used in this study.

sgRNA	Sequence (5'-3') (sgRNA+*PAM*)
sgRNA-MLS1	CAACTTCAACCAGCGACGTC *TGG* (134/fw)
sgRNA-CIT2	GCGATTTGCTTCTGACCTCA *AGG* (80/fw)
sgRNA-HPDH	GTCCTACGGCTTCATTGGTC *TGG* (86/fw)
sgRNA-MMSDH	TGCGAAAATCGAGAACCCCG *TGG* (101/fw)

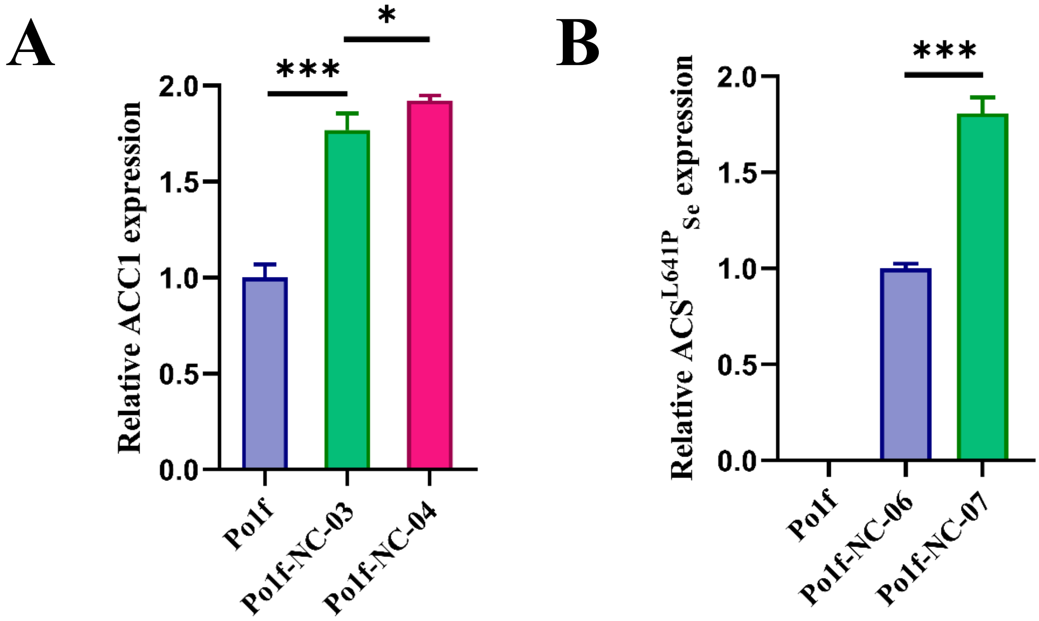

Figure S1. (A) The relative ACC1 expression of strain Po1f, Po1f-NC-03 and Po1f-NC-04. Statistical analysis was conducted as significance indicated by ***$P < 0.001$, *$P < 0.05$. **(B)** The relative ACS$^{L641P}_{Se}$ expression of strain Po1f, Po1f-NC-06 and Po1f-NC-07. Statistical analysis was conducted as significance indicated by ***$P < 0.001$. Data represent the mean ± SD of biological triplicates in **A** and **B**.

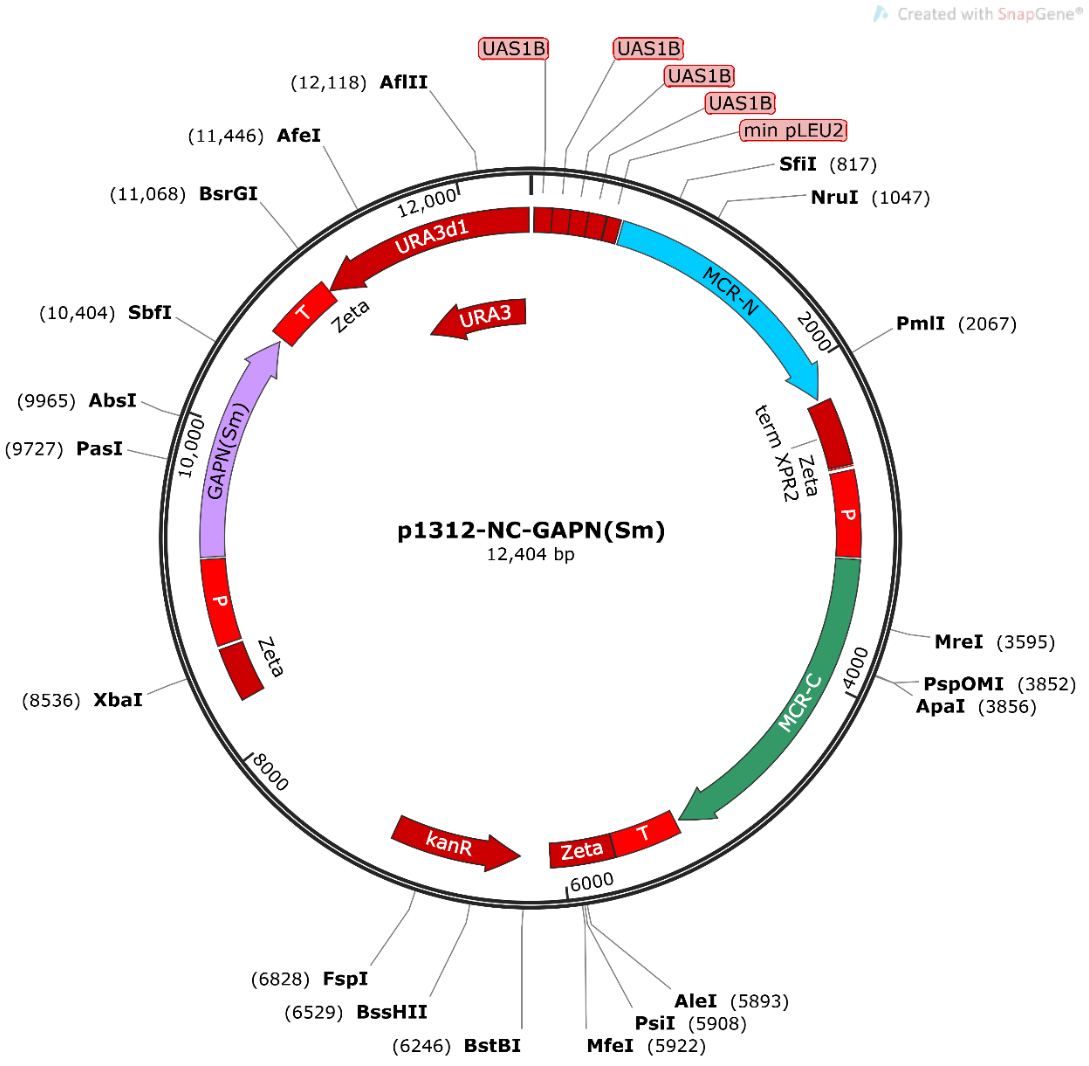

Figure S2. The map of plasmid p1312-NC-$GAPN_{Sm}$

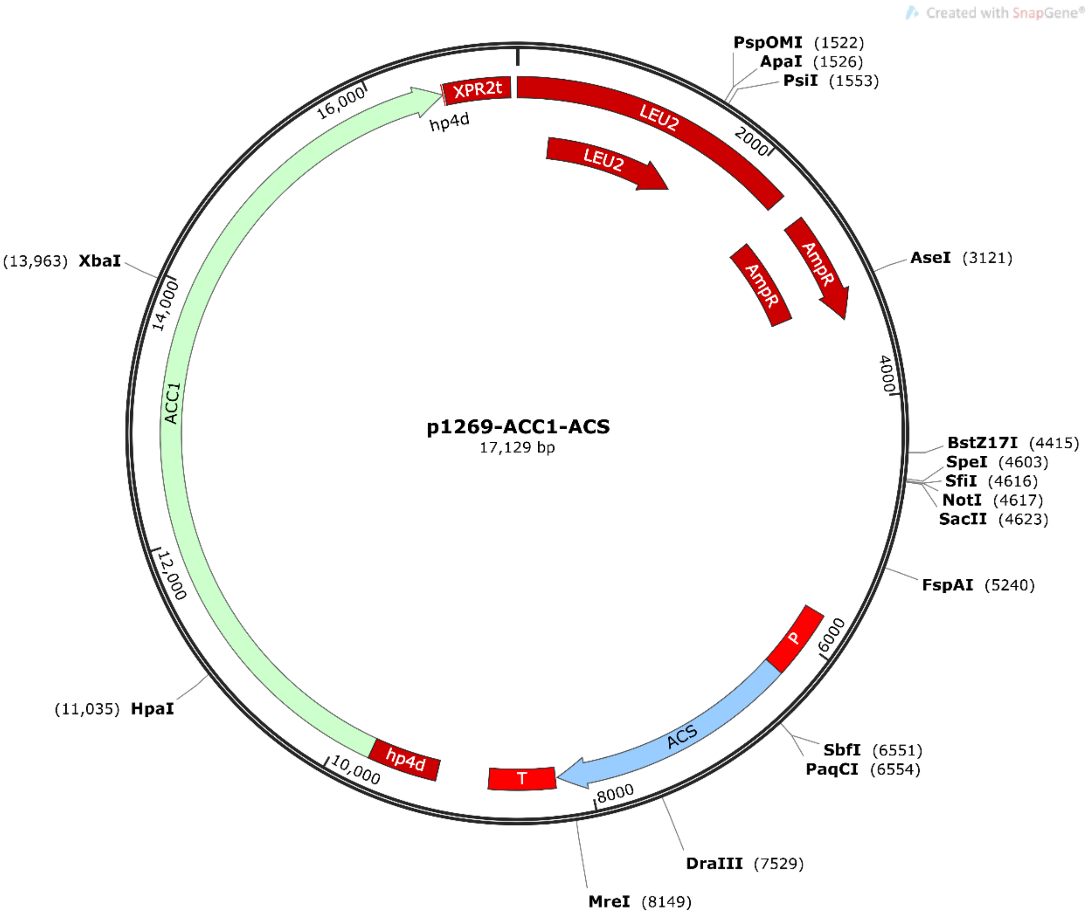

Figure S3. The map of plasmid p1269-ACC1-ACS$^{L641P}{}_{Se}$
